# Designing Trifluoromethyl Pyrazolones for Selective Molluscicidal Activity Against : Toward Sustainable Land Snail Control

**DOI:** 10.1021/acs.jafc.4c12327

**Published:** 2025-06-11

**Authors:** Hend M A Maaroof, Shaikha S. AlNeyadi, Yasir S. Raouf, Fatma I El-Akhrasy, Abdalla E. A. Hassan, Reham A I Abouelkhair

**Affiliations:** † Applied Nucleic Acids Research Center & Chemistry Department, Faculty of Science, 110193 Zagazig University, Zagazig 44519, Egypt; ‡ Department of Chemistry, College of Science, 11239 United Arab Emirates University, Al Ain 15551, United Arab Emirates; § Plant Protection Research Institute, Agricultural Research Center, Dokki, Giza 12622, Egypt

**Keywords:** terrestrial gastropods, land snail control, phenyl-pyrazolone, molluscicidal agents, induced fit docking, GABAA-GluCl blockers

## Abstract

Terrestrial gastropods are significant agricultural pests and disease carriers, posing major challenges to farm crops and various agricultural domains. Synthetic pesticides remain the primary method of pest control, but rising resistance and harmful effects on nontarget species highlight the urgent need for new, safer alternatives. Herrin, we report on the synthesis and the evaluation of a series of 5-trifluoromethyl-phenylpyrazolones against (O. F. Müller, 1774) snails as potential molluscicidal agents. The newly synthesized 5-trifluoromethyl-phenyl pyrazolones derivatives **11–24** were characterized based on ^1^H NMR, ^13^C-APT and ^19^F- NMR spectra as well as mass spectroscopy. Compounds **11**, **16**, **17** and **18** demonstrated potent anti- activities, with **16** exhibiting the highest lethal activity (LC_50_ = 0.58 mg/mL), surpassing the current standard, methomyl (LC_50_ = 2.28 mg/mL). Significant increase in liver transaminase enzymes (AST and ALT), acetylcholinesterase (AChE), along with reduction in total carbohydrates and lipids, were observed after the treatment of snails with compounds **11**, **16**, **17**, and **18** at the LC_50_ values. Histopathological analysis of the digestive glands of treated snails revealed induced cellular damage, with the greatest structural cellular integrity loss and functional impairment observed for compound **16**. Additionally, typical CNS toxicity symptoms, including paralysis and excessive fluid secretion, suggest a dual mode of action: gastrointestinal toxicity and GABA_A_-glutamate chloride ion channel (GluCl) antagonism. Homology modeling using the 3RHW ( (Maupas, 1900)) template and a GluCl ζ-subunit sequence were used to generate a 3RHW- ζ chimera for virtual screening. Additionally, induced fit docking (IFD) studies using GABA_A_ GluCl structures were used to generate trifluoromethylphenylpyrazole-compatible binding pockets. Docking scores derived from these models were found to support the observed molluscicidal activity. These findings identified potent 4-substituted-5-trifluoromethylphenylpyrazolones as potential useful candidates for safer, effective molluscicides.

## Graphical abstract

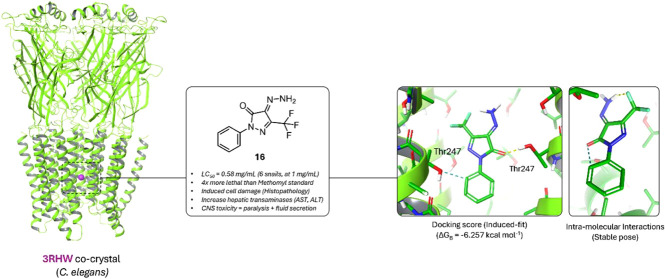

## 1 Introduction

Mollusks represent the second-largest phylum in the animal kingdom, consisting of seven classes: Aplacophora, Monoplacophora, Polyplacophora, Bivalvia, Gastropoda, Scaphopoda, and Cephalopoda.^1,2^ Among these, Gastropoda is the most diverse group, comprising over 100,000 species. Many gastropod species pose significant threats to agriculture, crops, and farming via serving as intermediate hosts for parasitic diseases that affect humans, animals, and plants.^3^ Over the past three decades, economic decline caused by just 13 invasive gastropod species has been estimated at $3.94 billion USD.^4^ (O. F. Müller, 1774) land snail is a terrestrial gastropod has been reported in many countries including Eastern and Western Europe,^5^ German, France, Ukraine,^5^ Poland,^6^ Czech Republic,^7^ and Iraq.^8^ land snails are characterized by a strong appetite, rapid reproduction, and exceptional capacity for distribution, factors that collectively cause severe reductions in crop yields and substantial economic losses for many farmers in different regions of the world. In addition, land snails are intermediate hosts for many parasites including *Brachylaima spp*., *Taenia spp*., lung worm spp., and metacercaria.^9^ In Egypt, favorable climates and annual weather conditions strongly facilitate the survival and proliferation of , making it a serious endemic agricultural pest.^10,11^ Effective pest control strategies are crucial for mitigating crop losses, enhancing food production, and supporting the Sustainable Development Goal (SDG) of Zero Hunger. Methomyl, a broad-spectrum anticholinesterase insecticide belonging to the oxime carbamate class, has shown high efficacy as a molluscicide.^12^ However, due to the development of resistance in certain insect species and its toxicity to nontarget organisms, methomyl has been banned in many European countries.^13^ Although the use of synthetic insecticides remains the most effective strategy for mollusk management^14^ undesirable side effects of established insecticide classes (e.g., carbamates, organophosphates, organochlorines, pyrethroids) have intensified the need for improved, and safer pesticide classes. To address this need, newer chemicals are ideally designed to have controlled, predictable mechanisms of action while minimizing environmental risks, aligning with the SDG agenda. Among these promising alternatives, the phenyl pyrazole family represents a well-established class of insecticides that target γ-aminobutyric acid (GABA) receptors. GABA is a neurotransmitter that typically inhibits nerve impulses, regulating nervous system activity. Fipronil (**1**), a trifluoromethyl-phenyl pyrazole derivative, has been widely used since its introduction in 1993 as a broad-spectrum insecticide^15^ (Figure 1). It binds specifically to GABA-gated chloride channels on nerve cells, blocking chloride ions from entering these cells. This inhibition disrupts normal nerve function, causing the neurons to fire uncontrollably, leading to hyperexcitation, paralysis, and eventually the insect’s death. By blocking chloride ion flow and impairing the insect central nervous system, fipronil ultimately results in lethal overstimulation. This molecular recognition event results in hyperexcitation and, eventually, insect death.^16,17^ Interestingly, fipronil selectively targets insect GABA receptors over mammalian ones, offering a certain degree of specificity with respect to its toxicity to humans.^18,19^ Fipronil has also been shown to possess broad molluscicidal activity against several species of land snails.^20^ However, several decades of repeated use (and in many cases misuse) have led to a rise in resistance in many target pest species.^21–23^ Moreover, major metabolites have been detected in a wide range of environmental matrices, including water, fish,^24^ soil,^25^ plants,^26^ and animal products,^27^ which is undesirable. Consequently, the use of Fipronil has been highly restricted or entirely banned in certain applications across regions like Europe,^28^ the United States, and China.^29^ Given these concerns, many second generation Fipronil analogs such as Flufiprole (**2**), and Pyriprole (**3**) have been developed to circumvent these concerns (Figure 1A). In addition, Pyrolan (**4**) is a phenyl pyrazole derivative with insecticidal properties (Figure 1A). Furthermore, Penthiopyrad (**5**) is a trifluoromethyl pyrazole fungicide, features a 4-substituted pyrazole core scaffold, a common motif in several agrochemicals (Figure 1B). As part of our ongoing efforts to develop potent and safer molluscicidal agents,^30^ this study focuses on the synthesis and evaluation of a new series of 4-substituted-5-trifluoromethyl-phenylpyrazolones. We investigated their molluscicidal activity, biochemical and histopathological effects, and molecular docking against GABA_A_ GluCl receptors (Figure 2). The design strategy incorporates a C5–CF_3_ group, a C4 electrophilic center (e.g., *gem*-dihalogeno, substituted-imino, oxime, or exocyclic methylene) intended to align with nucleophilic sites on target proteins, and a C3 carbonyl group serving as a hydrogen bond acceptor—distinguishing these compounds from Fipronil-like analogs. Our objective is to identify structurally optimized candidates with enhanced target specificity and reduced off-target toxicity.

**Figure 1:**
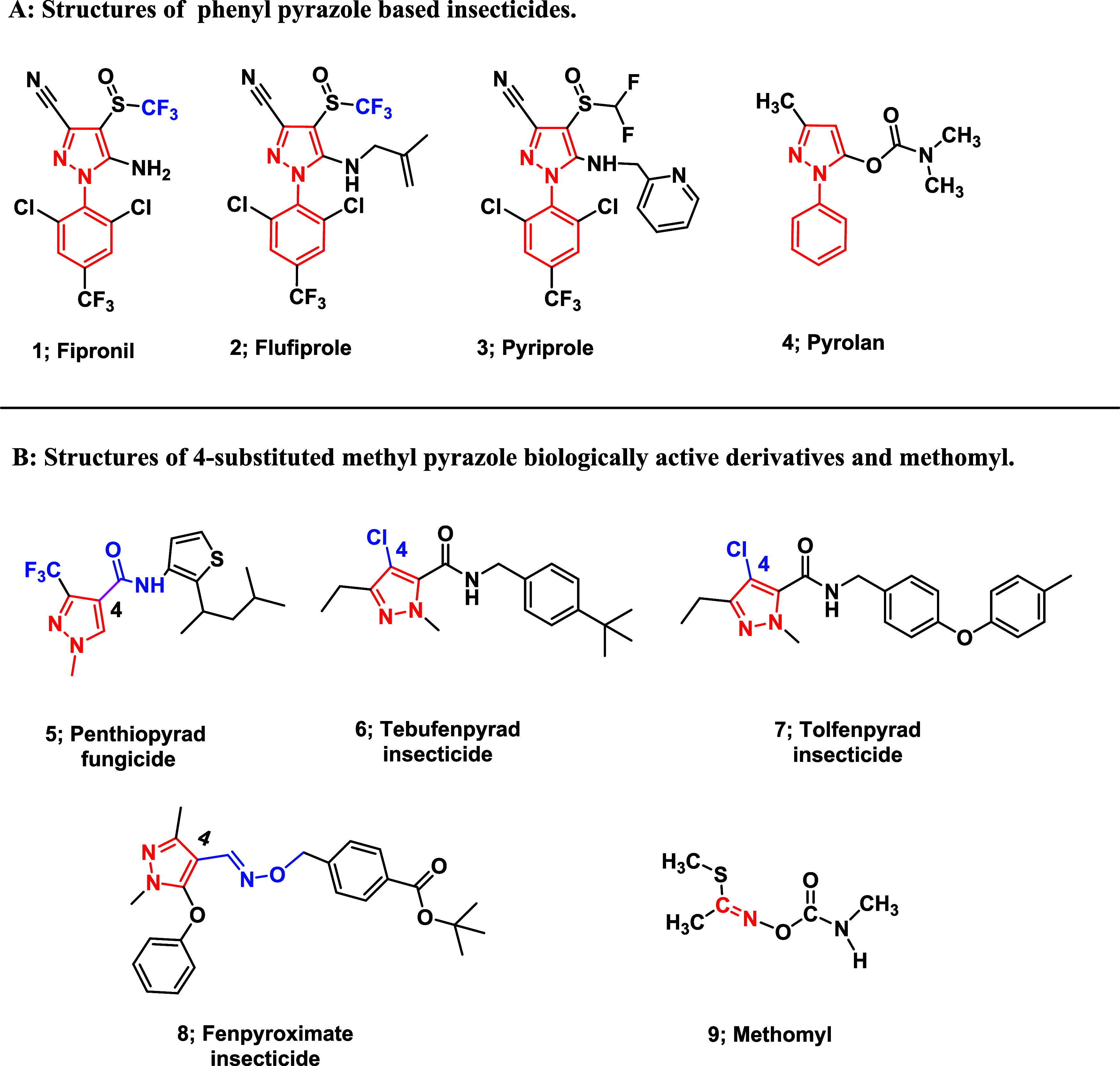
Structures of phenyl pyrazole insecticide derivatives, biologically active pyrazole derivatives and methomyl (standard molluscicide).

**Figure 2:**
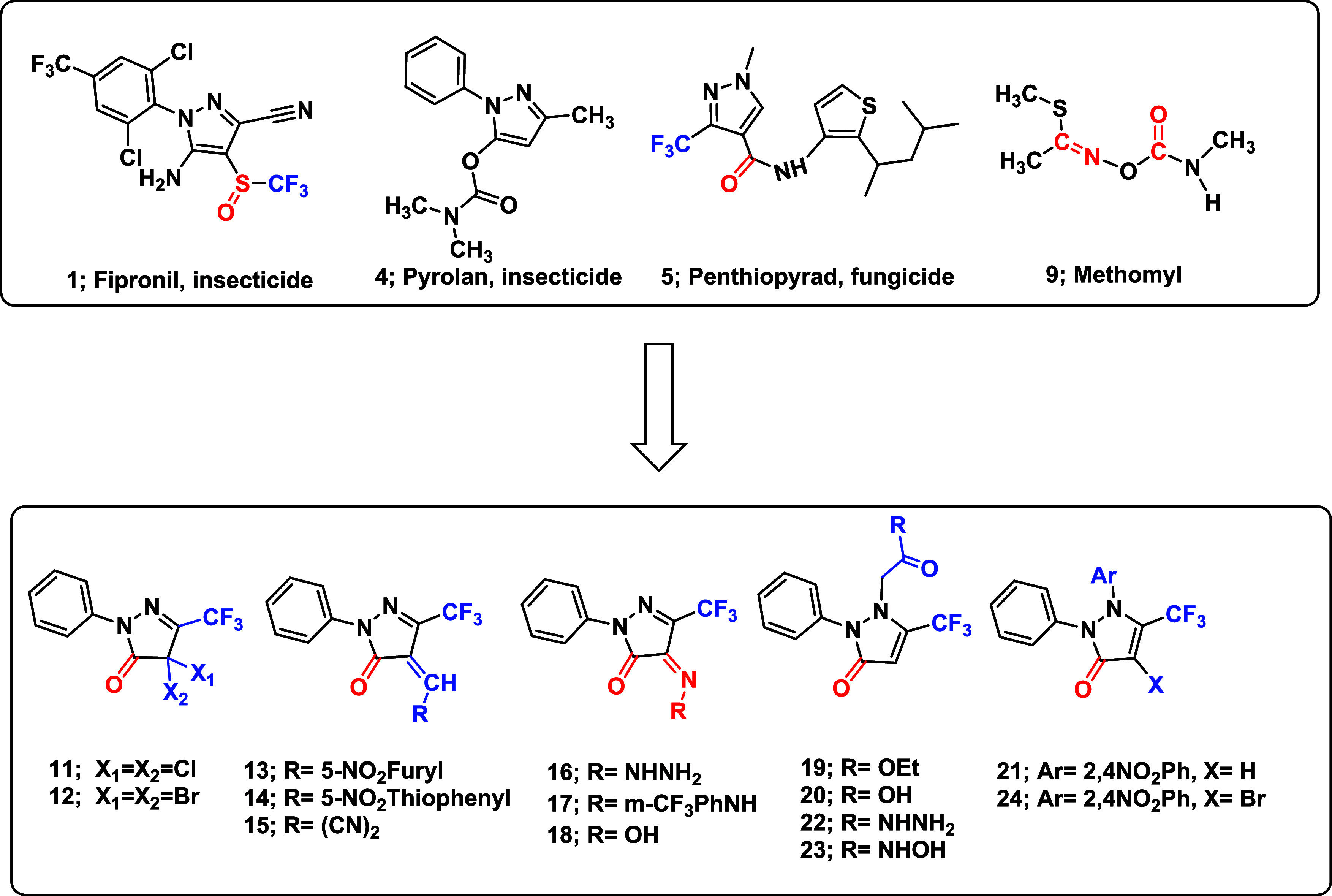
Schematic representation of the design strategy for modified 5-trifluoromethylphenylpyrazole derivatives **11–24**.

## 2 Materials and Methods

### 2.1 Chemistry

#### 2.1.1 General Methods

All chemicals were purchased from Sigma-Aldrich (St. Louis, MO) and used as received, without further purification. FTIR spectra were recorded on ATR-α-spectrometer (Bruker Corporation, Billerica, MA). Nuclear Magnetic Resonance (NMR) spectra, including ^1^H NMR, ^13^C-APT NMR, and ^19^F-NMR, were recorded on a Bruker 400 MHz spectrometer (Bruker Corporation, Billerica, MA) using deuterated solvents. Mass spectrometry was recorded on Agilent 6546 LC/Q-TOF (Agilent technologies, Santa Clara, CA).

##### 2-Phenyl-5-(trifluoromethyl)-1,2-dihydro-3*H*-pyrazol-3-one (**10**)

A solution of phenyl hydrazine hydrochloride (14.5 g, 0.1 mmol) and ethyl 4,4, 4, 4-trifluoroacetoacetate in glacial acetic acid (17.5 mL) was heated at reflux for 18 h. After cooling to room temperature, the solvent was concentrated under reduced pressure, and the resulting solid was filtered, to give **10** as a pale-yellow crystal (10 g, 73% yield). ^1^H NMR (400 MHz, DMSO-*d*_6_) δ: 12.46 (s, 1H, NH), 7.71 (d, *J* = 8.3 Hz, 2H, Ar), 7.51 (t, *J* = 7.9 Hz, 2H, Ar), 7.38 (t, *J* = 7.4 Hz, 1H, Ar), 5.94 (s, 1H, H-4); ^13^C NMR (101 MHz, DMSO-*d*_6_) δ: 153.7 (CO), 140.44 (q, *J*_C–F_ = 37.3 Hz, C-5), 137.74 (Ar), 129.11 (Ar), 127.22 (Ar), 122.69 (q, J_C–F_ = 267 Hz, CF_3_), 122.28 (Ar), 85.63 (C-4); ^19^F-NMR (376 MHz, DMSO-*d*_6_) δ: −61.85 (s, CF_3_).

##### 4,4-Dichloro-2-phenyl-5-(trifluoromethyl)-2,4-dihydro-3H-pyrazol-3-one (**11**)

A solution of compound **10** (200 mg, 0.881 mmol) and *N*-chlorosuccinimide (234 mg, 3.5 mmol) in dry THF (10 mL) was stirred at room temperature overnight. The solvent was evaporated under reduced pressure, and the residue was purified by column chromatography (ethyl acetate: hexane) to give **10** as yellow crystals (244 mg, 94.2%). MS ESI *m*/*z* 262.5 [M-CH_3_OH-H]^−^; HRMS ESI *m*/*z* 296.98 [M + H]^+^; ^1^H NMR (400 MHz, CDCl_3_) δ: 7.79 (d, *J* = 8 Hz, 2H, Ar), 7.47 (t, *J* = 12 Hz, 2H, Ar), 7.33 (t, *J* = 8 Hz, 1H, Ar); ^13^C NMR (101 MHz, CDCl_3_) δ: 163.27 (CO), 144.24 (C-5, *J*_C–F_ = 39 Hz), 135.89 (Ar), 129.53 (Ar), 127.62 (Ar), 119.48 (Ar), 116.80 (CF_3_, J_C–F_ = 272 Hz), 67.84 (C-4); ^19^F-NMR (376 MHz, CDCl_3_) δ: −63.59 (CF_3_).

##### 4,4-Dibromo-2-phenyl-5-(trifluoromethyl)-2,4-dihydro-3H-pyrazol-3-one (**12**)

A solution of compound **10** (250 mg, 1.1 mmol) and *N*-bromosuccinimide (390 mg, 2.2 mmol) in dry THF (10 mL) was stirred for 1 h at room temperature. The solvent was evaporated under reduced pressure, and the residue was purified by column chromatography (ethyl acetate: hexane) to give **12** as a dark green solid (400 mg, 94.3%). MS ESI *m*/*z* 468.9 [M + 2CHCN + H]^+^;^1^H NMR (400 MHz, CDCl_3_) δ: 7.83–7.80 (m, 2H, Ar), 7.48 (t, *J* = 8 Hz, 2H, Ar), 7.34 (t, *J* = 8 Hz, 1H, Ar); ^19^F-NMR (376 MHz, CDCl_3_) δ: −62.79 (CF_3_).

##### (*Z*)-4-((5-Nitrofuran-2-yl)­methylene)-2-phenyl-5-(trifluoromethyl)-2,4-dihydro-3H-pyrazol-3-one (**13**)

To a solution of compound **10** (200 mg, 0.881 mmol) and 5-nitro-2-furaldehyde diacetate (213 mg, 0.881 mmol) in glacial acetic acid (3.5 mL), 88 μL of conc. H_2_SO_4_ was added. The reaction mixture was stirred overnight at room temperature. The resulting solid was filtered and washed with distilled water to give **13** as red crystals (250 mg, 80.7%). MS ESI *m*/*z* 352.7 [M + H]^+^; ^1^H NMR (400 MHz, CDCl_3_) δ: 8.87 (d, *J* = 4.0 Hz, 1H, H-furan ring), 7.88 (d, *J* = 8 Hz, 2H, Ar), 7.64 (s, 1H, H-furan ring), 7.50–7.46 (m, 3H, Ar and H-olefinic), 7.32 (t, *J* = 8 Hz, 1H, Ar); ^13^C NMR (101 MHz, CDCl_3_) δ: 160.73 (CO), 150.28 (Ar), 139.80 (q, *J*_C–F_ = 38.5 Hz), 137.08 (Ar), 129.60 (CH, olefinic), 129.29 (Ar), 126.93 (Ar), 126.51 (Ar), 126.05 (Ar), 122.41 (Ar), 120.89 (q, *J*_C–F_ = 269.7 Hz), 119.97 (Ar), 113.56 (Ar), 112.98 (Ar); ^19^F-NMR (376 MHz, CDCl_3_) δ: −63.86 (CF_3_).

##### (*Z*)-4-((5-Nitrothiophen-2-yl)­methylene)-2-phenyl-5-(trifluoromethyl)-2,4-dihydro-3H-pyrazol-3-one (**14**)

To a solution of compound **10** (200 mg, 0.881 mmol) and 5-nitrothiophene-2-carboxaldehyde (204 mg, 0.881 mmol) in glacial acetic acid (3.5 mL), 88 μL of conc. H_2_SO_4_ was added. The reaction mixture was stirred overnight at room temperature. The solid product was filtered and washed with distilled water to give **14** as a red crystal (300 g, 92.7%). MS ESI *m*/*z* 412.8 [M + 2Na–H]^+^; ^1^H NMR (400 MHz, CDCl_3_) δ: 7.99 (d, *J* = 4.4 Hz, 1H, H-thiophene ring), 7.92 (dd, *J* = 8.7, 1.0 Hz, 2H, Ar), 7.86 (s, 1H, H-olefinic), 7.82 (d, *J* = 4.4 Hz, 1H, H-thiophene ring), 7.53–7.43 (m, 2H, Ar), 7.31 (dd, *J* = 10.7, 4.2 Hz, 1H, Ar); ^13^C NMR (101 MHz, CDCl_3_) δ: 161.29 (CO), 159.23 (Ar), 140.82 (CH, olefinic), 139.89 (Ar), 139.42 (q, *J*_C–F_ = 38 Hz, C-5), 138.15 (Ar), 137.13 (Ar), 129.35 (Ar), 127.52 (Ar), 126.90 (Ar), 120.63 (Ar), 119.83 (Ar), 119.74 (q, *J*_C–F_ = 270 Hz, CF_3_); ^19^F-NMR (376 MHz, CDCl_3_) δ: −63.57 (CF_3_).

##### 2-(5-Oxo-1-phenyl-3-(trifluoromethyl)-1,5-dihydro-4H-pyrazol-4-ylidene)­malononitrile (**15**)

To a solution of compound **12** (100 mg, 0.336 mmol) and malononitrile (33 mg, 0.504 mmol) in dry acetonitrile (CH_3_CN), 93 μL of triethylamine (Et_3_N) was added dropwise at 0 °C. The reaction mixture was stirred for 30 min, after which the solvent was evaporated under reduced pressure. The residue was diluted with ethyl acetate and washed with brine three times. The organic phase was dried over anhydrous sodium sulfate, filtered, and evaporated under reduced pressure. The product was purified by column chromatography (ethyl acetate: hexane) to give **15** as a yellow syrup (65 mg, 67%). MS ESI *m*/*z* 273.8 [M-H_2_O + H]^+^; ^1^H NMR (400 MHz, CDCl_3_) δ: 7.68 (d, *J* = 7.6 Hz, 1H, Ar), 7.60 (d, *J* = 7.6 Hz, 1H, Ar), 7.51–7.39 (m, 3H, Ar). ^13^C NMR (101 MHz, CDCl_3_) δ 156.58 (CO), 136.87 (q, *J*_C–F_ = 39 Hz, C-5), 130.25 (Ar), 129.36 (Ar), 128.52 (Ar), 125.01 (Ar), 123.43 (Ar), 120.64 (q, *J*_C–F_ = 269 Hz,), 111.73 (2 CN), 91.47 (C (CN)).

##### (*Z*)-4-Hydrazineylidene-2-phenyl-5-(trifluoromethyl)-2,4-dihydro-3H-pyrazol-3-one (**16**)

To a solution of compound **12** (200 mg, 0.672 mmol) in THF, 168 μL of hydrazine hydrate was added dropwise at 0 °C. The mixture was stirred for 30 min, and the solvent was evaporated under reduced pressure. The residue was diluted with ethyl acetate and washed with brine three times. The organic phase was dried over anhydrous sodium sulfate, filtered, and evaporated under reduced pressure. The residue was purified with flash column chromatography (ethyl acetate: hexane) to give **16** as a yellow solid (130 mg, 75.5%). MS ESI *m*/*z* 273.8 [M + NH_4_]^+^; ^1^H NMR (400 MHz, DMSO-*d*_6_) δ: 7.77 (d, *J* = 8.1 Hz, 2H, Ar), 7.55 (t, *J* = 7.7 Hz, 2H, Ar), 7.41 (t, *J* = 7.7 Hz, 1H, Ar), 3.51 (br s, exchangable with D_2_O, 2H, NH_2_).; ^13^C NMR (101 MHz, DMSO-*d*_6_) δ 152.91 (CO), 151.48 (C-4), 140.35 (Ar), 137.61 (q, *J*_C–F_ = 40.3 Hz. C-5), 129.22 (Ar), 127.98 (Ar), 127.25 (Ar), 122.89 (Ar), 122.15 (Ar), 119.27 (q, *J*_C–F_ = 264 Hz. CF_3_). ^19^F-NMR (376 MHz, DMSO-*d*_6_) δ: −61.37 (CF_3_).

##### (*Z*)-2-Phenyl-5-(trifluoromethyl)-4-((3-(trifluoromethyl)­phenyl)­diazenyl)-1,2-dihydro-3H-pyrazol-3-one (**17**)

To a cold solution of compound **10** (1 g, 4.4 mmol) in ethanol, sodium acetate (0.721 g, 8.8 mmol) was added, followed by dropwise addition of *m*-trifluoromethyl aniline diazonium salt (6.6 mmol) at 0 °C. The reaction mixture was stirred at room temperature for an hour. The precipitate was filtered, washed with cold water, and recrystallized from ethanol to give compound **17** (1.7 g, 97.8%) as yellow crystals. MS ESI *m*/*z* 369.5 [M-CH_3_OH + H]^+^; HRMS ESI *m*/*z* 399.06 [M-H]^−^; ^1^H NMR (400 MHz, CDCl_3_) δ: 13.95 (s, 1H, NH), 7.97–7.87 (m, 2H, Ar), 7.75–7.65 (m, 2H, Ar), 7.60 (t, *J* = 7.8 Hz, 1H, Ar), 7.55 (d, *J* = 7.7 Hz, 1H, Ar), 7.52–7.43 (m, 2H, Ar), 7.31 (t, *J* = 7.4 Hz, 1H, Ar); ^13^C NMR (101 MHz, DMSO-*d*_6_) δ 155.29 (CO), 142.40 (Ar), 137.19 (Ar), 131.07 (Ar), 130.32 (q, *J*_C–F_ = 32.3 Hz, C-5), 129.29 (Ar), 126.34 (Ar), 123.91 (Ar), 123.04 (q, *J*_C–F_ = 270 Hz, CF_3_), 120.87 (Ar), 119.14 (Ar), 114.11 (Ar). ^19^F-NMR (376 MHz, CDCl_3_) δ: −63.02 (s, CF_3_), −64.26 (s, CF_3_).

##### 4-(Hydroxylimino)-3-(trifluoromethyl)-1H-pyrazol-5­(4H)-one (**18**)

Nitrous acid, generated in situ by the addition of hydrochloric acid to sodium nitrite, was passed into a solution of compound **10** (1 g, 10.20 mmol) in methanol at room temperature for 18 h. The methanol was concentrated under vacuum, and the resulting solid was filtered and washed with cold methanol to give **18** (1.2 g, 93%) as yellow crystals. MS ESI *m*/*z* 301.6 [M + 2Na–H]^+^; HRMS ESI *m*/*z* 256.03 [M-H]^−^;^1^H NMR (400 MHz, DMSO-*d*_6_) δ: 7.81–7.68 (m, 2H, Ar), 7.59–7.37 (m, 2H, Ar), 7.38–7.26 (m, 1H, Ar). ^13^C NMR (101 MHz, DMSO-*d*_6_) δ 159.10 (CO, *Z*-isomer), 150.37 (CO, *E*-isomer), 140.42 (C-4, *Z*-isomer), 139.26 (C-4, *E*-isomer), 136.75 (q, *J* = 38 Hz, 2C-5), 129.15 (Ar, *Z*-isomer), 129.11 (Ar, *E*-isomer), 126.54 (Ar, *Z*-isomer), 126.42 (Ar, *E*-isomer), 119.75 (Ar, *Z*-isomer), 119.66 (Ar, *E*-isomer), 119.15 (d, *J*_C–F_ = 276 Hz, CF_3_, *Z*-isomer), 119.05 (d, J_C–F_ = 269 Hz, CF_3_, *E*-isomer). ^19^F NMR (376 MHz, DMSO-*d*_6_) δ −64.18 (CF_3_, *Z*-isomer), −65.52 (CF_3_, *E*-isomer).

##### General Procedure for the Synthesis of *N* ^1^-Substituted Pyrazolone Derivatives

A mixture of compound **10** (200 mg, 0.881 mmol), an alkylating agent (namely, 2,4-dinitrochlorobenzene, bromoacetic acid, or ethyl bromoacetate, 1.5 equiv), and dry potassium carbonate (K_2_CO_3_, 1.5 equiv) was suspended in dry acetone (15 mL) and heated at reflux for 12 h. The solvent was evaporated *in vacuo*, and the residue was purified using column chromatography.

##### Ethyl-2-(3-oxo-2-phenyl-5-(trifluoromethyl)-2,3-dihydro-1H-pyrazol-1-yl) acetate (**19**)

Elution with 10% ethyl acetate: hexane to give **19** as a yellow syrup (240 mg, 88%). MS ESI *m*/*z* 336.7 [M + Na]^+^; 312.7 [M-H]^−^; ^1^H NMR (400 MHz, CDCl_3_) δ: 7.76 (d, *J* = 8 Hz, 1H, Ar), 7.46 (t, *J* = 8 Hz, 1H, Ar), 7.36 (t, *J* = 8 Hz, 1H, Ar), 5.91 (s, 1H, H-4), 4.70 (s, 1H, CH_2_), 4.29 (d, *J* = 8 Hz, 1H, CH_2_), 0.33 (s, 3H, CH_3_); ^13^C NMR (101 MHz, CDCl_3_) δ: 167.07 (CO), 153.87 (CO), 142.17 (q, *J*_C–F_ = 39 Hz, C-5), 139.46 (Ar), 137.69 (Ar), 129.21 (Ar), 127.98 (Ar), 123.26 (Ar), 122.39 (q, *J*_C–F_ = 267 Hz, CF_3_), 85.35 (C-4), 68.66 (CH_2_), 62.15 (CH_2_), 14.30 (CH_3_); ^19^F-NMR (376 MHz, CDCl_3_) δ: −63.27 (s, CF_3_).

##### 2-(3-Oxo-2-phenyl-5-(trifluoromethyl)-2,3-dihydro-1H-pyrazol-1-yl) acetic acid (**20**)

Elution with 15% ethyl acetate: hexane to give **20** as a yellow syrup (105 mg, 41.8%). MS ESI *m*/*z* 330.6 [M + 2Na–H]^+^; ^1^H NMR (400 MHz, DMSO-*d*_6_) δ: 13.18 (s, 1H, OH), 7.75–7.73 (m, 2H, Ar), 7.55 (t, *J* = 8 Hz, 2H, Ar), 7.43 (t, *J* = 8 Hz, 1H, Ar), 6.52 (s, 1H, H-4), 4.95 (s, 2H, CH_2_); ^13^C NMR (101 MHz, DMSO-*d*_6_) δ: 168.93 (CO), 154.35 (CO), 140.40 (q, *J*_C–F_ = 37.8 Hz, C-5), 137.16 (Ar), 129.26 (Ar), 127.93 (Ar), 124.10–123.46 (m), 122.83 (Ar), 121.12 (q, *J*_C–F_ = 267 Hz, C-5), 86.10 (C-4), 68.33 (CH_2_); ^19^F-NMR (376 MHz, DMSO-*d*_6_) δ: −61.85 (CF_3_).

##### 1-(2,4-Dinitrophenyl)-2-phenyl-5-(trifluoromethyl)-1,2-dihydro-3H-pyrazol-3-one (**21**)

Elution with 5% ethyl acetate: hexane to give **21** as a yellow syrup (120 mg, 34.5%). MS ESI *m*/*z* 412.8 [M + NH4]^+^; ^1^H NMR (400 MHz, CDCl_3_) δ: 8.85 (d, *J* = 2.5 Hz, 1H, Ar), 8.41 (dd, *J* = 9.1, 2.5 Hz, 1H, Ar), 7.67 (d, *J* = 8.1 Hz, 2H, Ar), 7.47 (t, *J* = 7.7 Hz, 2H, Ar), 7.43–7.36 (m, 1H, Ar), 7.32 (d, *J* = 9.2 Hz, 1H, Ar), 6.32 (s, 1H, H-olefinic); ^13^C NMR (101 MHz, CDCl_3_) δ: 152.71 (CO), 147.40 (Ar), 143.70 (Ar), 142.64 (q, *J*_C–F_ = 39.6 Hz, C-5), 139.65 (Ar), 136.49 (Ar), 130.80 (Ar), 129.75 (Ar), 129.54 (Ar), 129.25 (Ar), 128.82 (Ar), 123.36 (Ar), 122.57 (Ar), 121.91 (q, *J*_C–F_ = 268 Hz, CF_3_), 119.12 (Ar), 92.66 (C-4); ^19^F-NMR (376 MHz, CDCl_3_) δ: −63.23 (s, CF_3_).

##### 2-(3-Oxo-2-phenyl-5-(trifluoromethyl)-2,3-dihydro-1H-pyrazol-1-yl)­acetohydrazide (**22**)

A solution of compound **19** (250 mg, 0.796 mmol) and hydrazine hydrate (200 mg, 3.98 mmol) in ethanol (10 mL) was heated at reflux for 12 h. The solvent was evaporated *in vacuo*, and the residue was diluted with ethyl acetate and washed with water. The organic layer was dried over anhydrous sodium sulfate, filtered, and evaporated under reduced pressure, then crystallized from methanol to give **22** as white crystals. MS ESI *m*/*z* 322.7 [M + Na]^+^; ^1^H NMR (400 MHz, DMSO-*d*_6_) δ: 9.41 (s, 1H, NH), 7.74 (d, *J* = 8.0 Hz, 2H, Ar), 7.54 (t, *J* = 7.7 Hz, 2H, Ar), 7.43 (s, 1H, Ar), 6.39 (s, 1H, H-4), 4.74 (s, 2H, CH_2_), 4.38 (s, 2H, NH_2_); ^13^C NMR (101 MHz, DMSO-*d*_6_) δ: 165.69 (CO), 154.61 (CO), 140.27 (q, *J*_C–F_ = 37.8 Hz, C-5), 137.23 (Ar), 129.50 (Ar), 127.90 (Ar), 122.89 (Ar), 122.89 (q, *J*_C–F_ = 267 Hz, CF_3_), 85.68 (C-4), 69.44 (CH_2_); ^19^F-NMR (376 MHz, DMSO-*d*_6_) δ: −61.81 (CF_3_).

##### *N*-Hydroxy-2-(3-oxo-2-phenyl-5-(trifluoromethyl)-2,3-dihydro-1H-pyrazol-1-yl)­acetamide (**23**)

A solution of compound **19** (200 mg, 0.557 mmol) and hydroxylamine hydrochloride (774 mg, 11.142 mmol) in dry pyridine (10 mL) was heated at reflux for 12 h. The solvent was evaporated under reduced pressure, and the residue was purified by column chromatography to give **23** as a dark red syrup (162 mg, 61%). ^1^H NMR (400 MHz, DMSO-*d*_6_) δ: 8.61 (s, 1H, NH), 7.74 (d, *J* = 7.5 Hz, 2H, Ar), 7.54 (t, *J* = 7.8 Hz, 2H, Ar), 7.44 (d, *J* = 7.3 Hz, 1H, Ar), 6.52 (s, 1H, H-4), 4.95 (s, 3H, OH, CH2); ^13^C NMR (101 MHz, DMSO) δ: 168.95 (CO), 154.37 (CO), 140.44 (q, JC = 37.8 Hz, C-5), 137.04 (Ar), 129.27 (Ar), 127.93 (Ar), 122.84 (Ar), 121.14 (q, *J*_C–F_ = 268 Hz, CF_3_), 86.12 (C-4), 68.36 (CH_2_); ^19^F-NMR (376 MHz, DMSO-*d*_6_) δ: −61.87 (CF_3_).

##### 4-Bromo-1-(2,4-dinitrophenyl)-2-phenyl-5-(trifluoromethyl)-1,2-dihydro-3H-pyrazol-3-one (**24**)

A solution of compound 21 (200 mg, 0.507 mmol) and *N*-bromosuccinimide (179 mg, 1.01 mmol) in glacial acetic acid (7 mL) was heated at reflux for 48 h. The solvent was evaporated *in vacuo*, and the residue was purified by column chromatography to give **24** as a yellow syrup (150 mg, 53.5%). ^1^H NMR (400 MHz, CDCl_3_) δ: 8.88 (d, *J* = 2.7 Hz, 1H, Ar), 8.39 (dd, *J* = 9.2, 2.7 Hz, 1H, Ar), 7.71–7.61 (m, 2H, Ar), 7.54–7.37 (m, 3H, Ar), 7.05 (d, *J* = 9.2 Hz, 1H, Ar); ^13^C NMR (101 MHz, CDCl3) δ: 151.68 (CO), 144.90 (Ar), 143.55 (Ar), 141.47 (q, *J*_C–F_ = 38.6 Hz, C-5), 136.35 (Ar), 130.00 (Ar), 129.76 (Ar), 129.48 (Ar), 122.94 (Ar), 122.78 (Ar), 112.44 (q, *J*_C–F_ = 269 Hz, CF_3_), 117.51 (C-4); ^19^F-NMR (376 MHz, CDCl_3_) δ: −63.29 (CF_3_).

### 2.2 Biological Studies

#### 2.2.1 Molluscicidal Activity

The molluscicidal activity of trifluoromethyl pyrazolone derivatives (compounds **11–24**) was evaluated against land snails under laboratory conditions, following the guidelines provided by the World Health Organization (WHO), with slight modifications.^31,32^ Methomyl and distilled water were used as control treatments for comparison. Detailed test procedures are provided in the Supporting Data. Snail mortality was determined by the lack of contraction response, and dead specimens were removed. Mortality rates were recorded after 24 h of exposure, and the LC_50_ values (lethal concentration for 50% of the population) were calculated using LdP line software “a software to calculate probit analyses according to Finney (1971)”.

#### 2.2.2 Biochemical Assays

The in vivo biochemical impact of selected trifluoromethyl pyrazolone compounds (**11**, **16**, **17**, and **18**) on snails was assessed. The activity of ALT, AST, AChE, total carbohydrates, and total lipids was measured according to standard biochemical methods^33–36^ For further details, see Section S2.2.1 in the Supporting Data.

#### 2.2.3 Histological Study

A histopathological study of the digestive gland sections of the land snails was conducted. They were observed by light microscopy after treatment with trifluoromethyl pyrazolone compounds **11**, **16**, **17**, and **18** compared with that of the no-treatment control. For further details, see Section S2.3.1 in the Supporting Data.

## 3 Results and Discussion

### 3.1 Chemistry

The starting material, 2-Phenyl-5-(trifluoromethyl)-1,2-dihydro-3*H*-pyrazol-3-one (**10**) was synthesized through the reaction of ethyl trifluoroacetoacetate and phenyl hydrazine hydrochloride in glacial acetic acid under reflux conditions.^37^ The structure of compound **10** was confirmed by ^1^H NMR spectroscopy, which revealed a singlet at δ 5.94 ppm corresponding to the olefinic proton, and a peak at δ 12.46 ppm for the NH proton. Additionally, five aromatic protons from the phenyl group were observed in the δ 7–8 ppm region. ^13^C NMR analysis showed two quaternary carbon signals at δ 140.44 and δ 122.69, attributed to C5 and the exocyclic CF_3_ group, respectively. The trifluoromethyl group was further confirmed by its ^19^F-NMR signal at δ −61.85 ppm. The halogenated trifluoromethyl pyrazolone derivatives, *gem*-dichloro (**11**) and *gem*-dibromo (**12**), derivatives were synthesized via the treatment of compound **10** with *N*-chlorosuccinimide (NCS) and *N*-bromosuccinimide (NBS),^38^ respectively, yielding the products in 94.2% and 94.3%, respectively. The structures of **11** and **12** were confirmed by the disappearance of the (H-4) olefinic proton in their ^1^H NMR spectra and the appearance of a quaternary carbon atom in their APT-^13^C NMR spectra. Appearance of the C-4 quaternary carbon atom of compound **11** at δ 67.84 ppm gives clear evidence that dihalo (*gem*) derivatives were formed. The reaction of 5-nitrofuraldehyde and 5-nitrothiophene with compound **10** at the C-4 active methylene under acidic conditions produced the corresponding α, β-unsaturated carbonyl compounds **13** and **14**, respectively in good yields (Scheme 1). The structure of compound **14** was confirmed by ^1^H NMR and APT-^13^C NMR spectra. ^1^H NMR spectrum of compound **14** showed the absence of the H-4 proton and the presence of two doublets at δ 7.99 and δ 7.82 ppm corresponding to the protons of the thiophene ring. The olefinic proton appeared as a singlet at δ 7.86 ppm. Treatment of compound **12** with malononitrile in the presence of triethylamine as a base resulted in the formation of the dicyanomethylene derivative **15** in moderate yield. ^1^H NMR spectrum of compound **15** confirmed the disappearance of the olefinic proton. Additionally, treating compound **12** with hydrazine hydrate at 0 °C produced the 4-hydrazon derivative **16** in good yield. However, when the reaction was performed at room temperature, compound **16** became a minor product, with compound **10** being the predominant species. The structure of compound **16** was thoroughly characterized using ^1^H NMR, ^13^C NMR, and ^19^F-NMR spectroscopy. The ^1^H NMR spectrum of compound **16** displayed a broad singlet at δ 3.5 ppm exchangeable with D_2_O, integrating for two protons corresponding to the NH_2_ group.

**Scheme 1:**
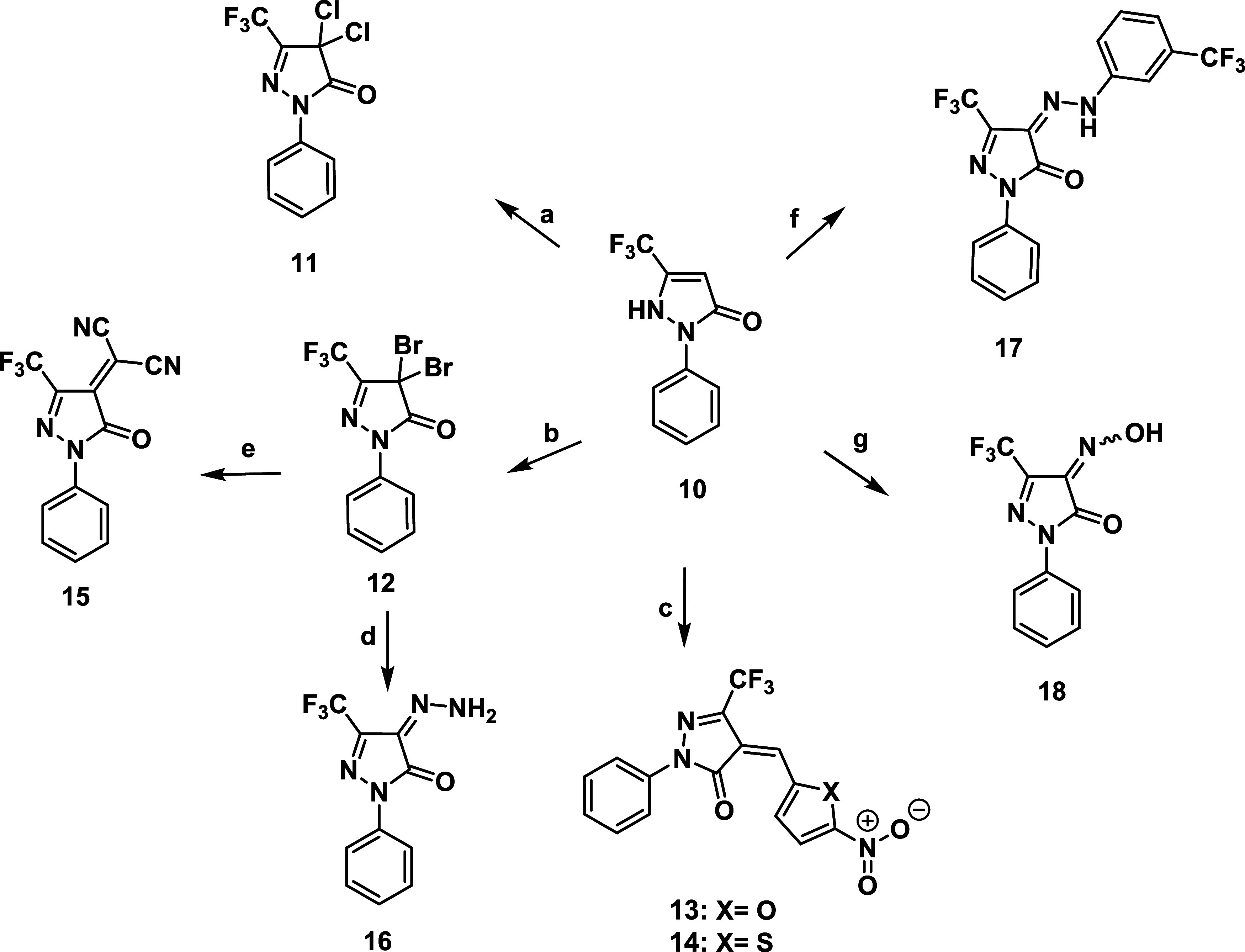
Reagents and Conditions^a^

a (a) NCS (4 equiv), THF, overnight, rt.; (b) NBS (2 equiv), THF, 1 h, rt.; (c) aldehyde (1 equiv), AcOH, conc. H_2_SO_4_ (catalyst); (d) CH_2_(CN)_2_ (1.5 equiv), CH_3_CN, Et_3_N, 1 h.; (e) NH_2_NH_3_^+^·OH^–^ (5 equiv), THF, 30 min, 0 °C; (f) *m*-trifluoromethylaniline, conc. HCl, NaNO_2_, AcONa, EtOH, 1 h.; (g) NaNO_2_, conc. HCl, MeOH, 18 h

Treatment of compound **10** with diazonium salts, generated from *m*-trifluoromethyl aniline in the presence of sodium acetate and absolute ethanol, afforded the corresponding 4-hydrazonopyrazolone derivative **17** in excellent yield^39^ (Scheme 1). The structure of compound **17** was isolated as a single isomer, confirmed by ^1^H, ^19^F, and ^13^C NMR spectroscopy. The ^1^H NMR spectrum of **17** displayed a singlet at δ 13.95 ppm, attributed to the NH group, along with signals in the aromatic region corresponding to the phenyl group. The ^19^F-NMR spectrum showed two peaks at δ −63.02 and −64.26 ppm, corresponding to the trifluoromethyl groups on the pyrazolone and phenyl rings, respectively. The 4-hydroxylimino derivative **18** was synthesized by nitrosation of compound **10** using sodium nitrite and concentrated HCl, following a slight modification of the procedure reported in the literature^40,41^ (Scheme 1). Compound **18** existed as Z, *E*-isomers of the hydroxyimine tautomer. This was clear in ^19^F, and ^13^C NMR spectra. ^19^F**-**spectrum showed two peaks at δ −64.18, −65.52 ppm corresponding to *Z* & *E*-isomers, respectively, and this is compatible with data reported by Burgart et al.^42^

The reaction of compound **10** with various organohalides in the presence of potassium carbonate (K_2_CO_3_) in refluxing acetone yielded the *N* ^1^-substituted phenylpyrazolone derivatives **19**, **20**, and **21** (Scheme 2). The structures of these compounds were confirmed by ^1^H, ^13^C, and ^19^F-NMR spectroscopy. For example, the ^1^H NMR spectrum of compound **21** showed a singlet at δ 6.32 ppm corresponding to H-4, and an increase in aromatic proton signals by 3 H-protons, indicating the presence of the phenyl ring. Refluxing compound **21** with NBS in glacial acetic acid resulted in the formation of the bromo derivative **24**, as evidenced by the disappearance of the ^1^H NMR signal for the olefinic H-4 proton and the absence of the CH peak in the APT-^13^C NMR spectrum, along with the appearance of a quaternary carbon at δ 117.51 ppm corresponding to C-4. Furthermore, the reaction of compound **19** with hydrazine hydrate in refluxing ethanol produced the acid hydrazide derivative **22** in good yield (Scheme 2). The ^1^H NMR spectrum of compound **22** exhibited a singlet at δ 9.41 ppm, corresponding to the NH group, and a signal at δ 4.74 ppm for the NH_2_ group. Additionally, the hydroxamic acid derivative **23** was synthesized by reacting compound **19** with hydroxylamine hydrochloride in refluxing pyridine. The ^1^H NMR spectrum of compound **23** confirmed the absence of the ethyl group.

**Scheme 2:**
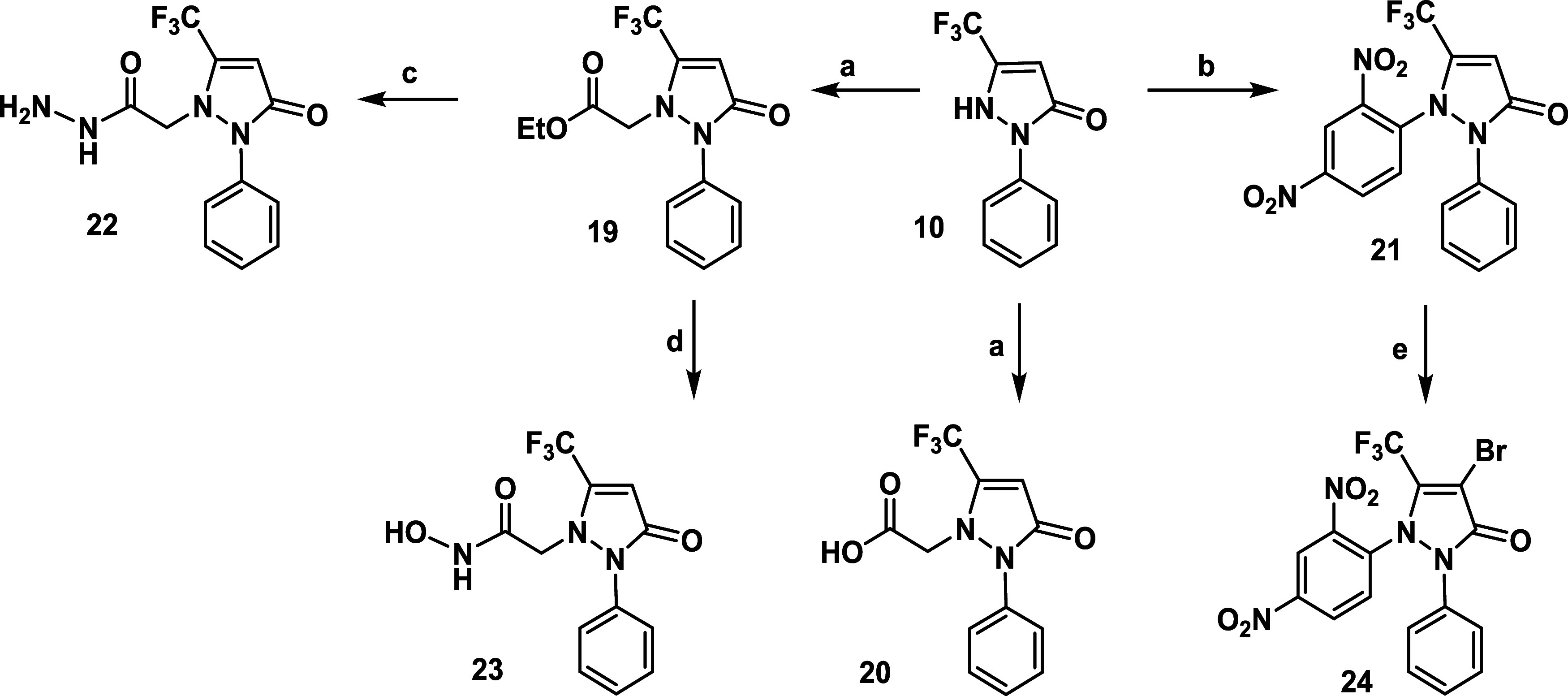
Reagents and Conditions^a^

a (a) appropriate alkylating agent (1.5 equiv), K_2_CO_3_ (1.5 equiv), acetone, reflux; (b) 2,4-dinitrochlorobenzene (1.5 equiv), K_2_CO_3_ (1.5 equiv), acetone, reflux; (c) NH_2_NH_3_^+^·OH^–^ (5 equiv), EtOH, reflux, 12 h.; (d) NH_2_OH·HCl (20 equiv), pyridine, reflux, 12 h.; (e) NBS (2 equiv), AcOH, reflux, 48 h.

### 3.2 Biology

#### 3.2.1 Molluscicidal Activity

The molluscicidal activity of the synthesized trifluoromethylpyrazolone derivatives **10**–**24** was preliminarily assessed against *M. cartusiana* snails. Methomyl, a commercially available molluscicide, was used as a positive control. Among the tested compounds, **11**, **16**, and **18** exhibited high mortality rates against . Whereas compound **17** exhibited moderate molluscicidal activity, and compounds **12**, **15**, **19**, **21**, and **23** demonstrated relatively weak molluscicidal activity. Compounds **13** and **14** were found to be devoid of any molluscicidal activity (Table 1). The LC_50_ values of compounds **11**, **16**, **17**, and **18** were determined (Table 1). The LC_50_ values of compounds **11**, **16**, **17**, and **18** are 1.93, 0.58, 3.8, and 1.11 mg/mL, respectively. Compound **16**, having a (C4) hydrazone moiety was found to surpass Methomyl, the positive control (LC_50_ = 2.28 mg/mL), while compounds **11**, having a C4 (*gem*-dichloro moiety) and **18** having C4 (oxime moiety) demonstrated comparable activity with that of methomyl. The structure–activity relationship (SAR) analysis of the tested compounds was clearly related to the functional group at the C4 position of the 5-trifluoromethylpyrazolone scaffold. Where the imino moiety at the C4-position (–C^4^=N–NH_2_ or –C^4^=N–OH) exhibited significantly higher molluscicidal activity compared with C^4^=N-NHAr, as in compound **17**. This indicates by increasing steric hindrance at the C4-hydrazone moiety by the incorporation of a bulky phenyl group dramatically reduced activity, by approximately 7.8-fold. Replacing the imino group (C^4^=N) with a vinyl moiety (C^4^=C) resulted in a complete loss of the molluscicidal activity as in compounds **13**, **14** and **15**. *gem*-C^4^–Cl_2_-5-Trifluoromethypyrazolone derivatives (**11)** displayed higher molluscicidal potency compared with the corresponding *gem*-C^4^–Br_2_, suggesting a favorable influence of chlorine on biological activity. In summary, compounds **11**, **16**, **17**, and **18** were identified as potent molluscicidal agents, with compound **16** exhibiting superior molluscicidal activity to that of Methomyl. These results highlight the importance of specific structural features, such as the imino moiety and minimal steric hindrance, in modulating the mollusicidal activity of trifluoromethylpyrazolone scaffold. Further optimization of these compounds could provide a basis for the development of more effective molluscicides targeting *M. cartusiana* land snails.

**Table 1:** Mollusicidal Activity and LC_50_ Values (mg/mL) of Compounds 11–24 and Methomyl (Positive Control) against Snails at Different Concentrations (1, 2, 3, and 4 mg/mL)^a^

	concentration				
compound	1 mg/mL	2 mg/mL	3 mg/mL	4 mg/mL	LC_50_ mg/mL
**11**	1 ± 0.5	6 ± 0.5	7 ± 0.5	9 ± 1.1	1.9 ± 0.31
**12**	0	1 ± 1	2 ± 0.5	2 ± 1	^b^
**13**	0	0	0	0	NA
**14**	0	0	0	0	NA
**15**	0	1 ± 0.5	1 ± 0.5	5 ± 1.1	
**16**	6 ± 1	7 ± 1	7 ± 0.5	8 ± 2	0.58 ± 0.14
**17**	3 ± 1.5	3 ± 0.5	4 ± 1	6 ± 0.5	3.8 ± 0.60
**18**	5 ± 1	7 ± 0.5	9 ± 1	9 ± 0.5	1.11 ± 0.35
**19**	3 ± 1.1	3 ± 0.0	3 ± 0.5	4 ± 1.5	
**20**	0	0	0	0	NA
**21**	0	3 ± 0.5	3 ± 0.5	5 ± 1.7	
**22**	0	0	1 ± 0.5	1 ± 0.5	
**23**	1 ± 0.5	3 ± 1	4 ± 1.5	5 ± 1.1	
**24**	1 ± 0.5	1 ± 1	1 ± 0.5	2 ± 1	
methomyl	1 ± 1	4 ± 0.5	5 ± 1	10 ± 0.0	2.28 ± 0.41

a The data represents the mean number ± SD of three replicates of snails killed from 10 individuals after 24 h of exposure. LC50 values represent the lethal concentrations required to kill 50% of the snail population. NA: not active.

b not calculated.

Symptoms observed on land snails upon treatment with compounds **11**, **16**, **17**, and **18** included excessive fluid secretions, internalization of the gastropod into the snail’s shell and ultimately death of the snail by the end 24 h’ treatment time. In other occasions, we have observed paralysis of the snail, manifested by exposure of the gastropod outside the snail’s shell and inability of the gastropod to internalize into the shell. Pinching the gastropod with a needle is encountered with no response from the snail, implying the snail is paralyzed (Supporting Data, Section 2.1). These typical CNS toxicity symptoms suggest a dual mode of action: gastrointestinal toxicity and GABA_A_-glutamate chloride ion channel (GluCl) antagonism. Therefore, the impact of compounds **11**, **16**, **17** and **18** on the gastrointestinal system (biochemical evaluation), snail’s digestive gland (histopathological evaluation), and induced fit docking (IFD) studies using GABA_A_ GluCl (CNS toxicity) were investigated.

#### 3.2.2 Biochemical Analysis

The biochemical impact of the trifluoromethylpyrazolone derivatives **11**, **16**, **17**, and **18** on various enzymatic and metabolic parameters in snails were evaluated (Table 2). The elevated levels of aspartate aminotransferase (AST) and alanine transaminase (ALT) following exposure to these compounds indicate significant hepatotoxic stress. AST and ALT are known biomarkers of liver function, and their increased levels suggest potential disruption of liver activity due to the compounds’ effects on detoxification and metabolic pathways. Compounds **16** and **18** demonstrated the highest increases in AST (1.67- and 1.53-fold, respectively) and ALT (1.17- and 1.11-fold, respectively), highlighting their potent biochemical activity. These observations are consistent with previous studies where pesticide exposure resulted in elevated AST and ALT levels in other organisms.^43,44^ The significant increase in these enzymes suggests that the tested compounds could induce oxidative stress, leading to liver damage and metabolic disturbances in . Similarly, the observed increases in acetylcholinesterase (AChE) activity (1.18- to 1.25-fold across the compounds) suggest interference with the nervous system’s functioning. While the increase is modest, it indicates a disruption in cholinergic neurotransmission, a common target for pesticides. The increase in AChE activity points to a potential compensatory mechanism by the snails to counteract the neurotoxic effects induced by the compounds. This observation supports the notion that trifluoromethylpyrazolones, like other neurotoxic agents, may exert molluscicidal activity through AChE inhibition. Interestingly, total carbohydrate levels were significantly reduced (2.43- to 3.68-fold), particularly in snails exposed to compound **16**, indicating metabolic disruption. The depletion of carbohydrate reserves could be attributed to the heightened metabolic demand or impaired glycolytic pathways, possibly due to liver dysfunction induced by the compounds. This reduction is further supported by the parallel increase in lipid levels, which may suggest a shift in energy metabolism from carbohydrates to lipids. Lipid accumulation, especially in response to compounds **16** and **18**, indicates altered lipid metabolism, which may be a compensatory response to the depletion of carbohydrate stores. The increased lipid levels (1.17- to 1.61-fold) suggest that these compounds could also be affecting lipid storage and utilization pathways in the snails. Taken together, these findings indicate that the trifluoromethyl pyrazolone derivatives not only exhibit molluscicidal activity but also cause significant biochemical perturbations in . The disruption of enzyme activities, depletion of carbohydrate reserves, and increase in lipid content suggest that these compounds exert their toxic effects through multiple metabolic pathways. The observed biochemical changes are indicative of the compounds’ ability to induce stress responses, impair metabolic functions, and disrupt energy homeostasis in the snails. These results highlight the impact of the trifluoromethylpyrazolone derivatives **11**, **16**, **17**, and **18** on disrupting metabolic enzymes as well as digestive system of the snails. Our findings are consistent with previous studies reporting that methomyl enhances the activities of aspartate transaminase (AST) and alanine transaminase (ALT) in , indicating tissue stress or damage.^12^ However, our results diverge in terms of methomyl’s effects on total lipid content and acetylcholinesterase (AChE) activity. Specifically, methomyl exerted a strong inhibitory effect on AChE activity in ,^45^ which aligns with its known mode of action as a carbamate insecticide that disrupts cholinergic neurotransmission. In addition, a marked reduction in total lipid content was observed on the first day of methomyl administration in both and .^46^

**Table 2:** Biochemical Parameters Measured in Land Snails after 24-h Exposure to the LC_50_ Values of Compounds **11**, **16**, **17**, and **18^a^**

compound no.	AST (mg/g/min)	ALT (mg/g/min)	AChE (mg/g/min)	Carb. (mg/g)	Lip (mg/g)
**control**	0.18 ± 0.01	41.8 ± 0.1	14.3 ± 0.1	97.1 ± 0.1	5.8 ± 0.8
**11**	0.24 ± 0.03	45.4 ± 0.6	16.8 ± 0.8	36.7 ± 0.9	8.1 ± 0.1
**16**	0.31 ± 0.01	49.1 ± 0.1	18.0 ± 2.0	26.4 ± 0.5	9.3 ± 0.1
**17**	0.23 ± 0.01	44.5 ± 0.1	16.7 ± 3.9	39.9 ± 3.7	6.8 ± 0.1
**18**	0.28 ± 0.01	46.8 ± 0.2	17.0 ± 1.8	35.4 ± 0.4	8.8 ± 0.1

a Values are mean ± SD of three replicates.

#### 3.2.3 Histopathological Study

The digestive gland is a vital organ in snails, responsible for digestion, absorption, and storage of nutrients, in addition to biotransformation of chemical and xenobiotic.^47^ The digestive gland of the control land snail, as represented in Figure 3A1,A2, consists of spherical tubules, which are separated by loose connective tissue containing lymphatic vessels and hemocytes. Each tubule is encased by a circular muscle layer. The epithelium lining the lumens of these tubules comprises several distinct cell types, including digestive cells, calcium cells, and excretory cells. Examination of the digestive gland sections of the tested land snail treated with compound **17** indicated degeneration between cells, resulted in a reduction in the size of both the excretory granules and calcium spherules (Figure 3B1). Additionally, the nuclei shift downward toward the center of the cells, while vacuoles become enlarged in the intercellular spaces. The connective tissue surrounding the gland acini begins to break down, resulting in fragmentation of the gland’s cellular architecture. This degeneration between cells causes the lumen to enlarge, accompanied by the dispersal of excretory granules within the lumen (Figure 3B2). Enlargement of vacuoles were observed in the digestive gland sections of the tested land snail treated with compound **11** (Figure 3 C1). Although the cell membranes maintain their structural integrity within the acini, the lumen continues to expand, and the nucleus migrated downward toward the cell apex. Furthermore, fragmentation of the cell basement membrane and disorientation of the nucleus was observed (Figure 3C2). The digestive gland sections of the tested land snail treated with **18** showed shrinking of the acini and leading to the enlargement of the surrounding connective tissue as well as appearing of small vacuoles (Figure 3D1). This treatment induced early degeneration within the acinic cells toward the lumen, where excretory granules begin to be released from the cells. Additionally, numerous vacuoles were formed around the basement membrane (Figure 3D2).

**Figure 3:**
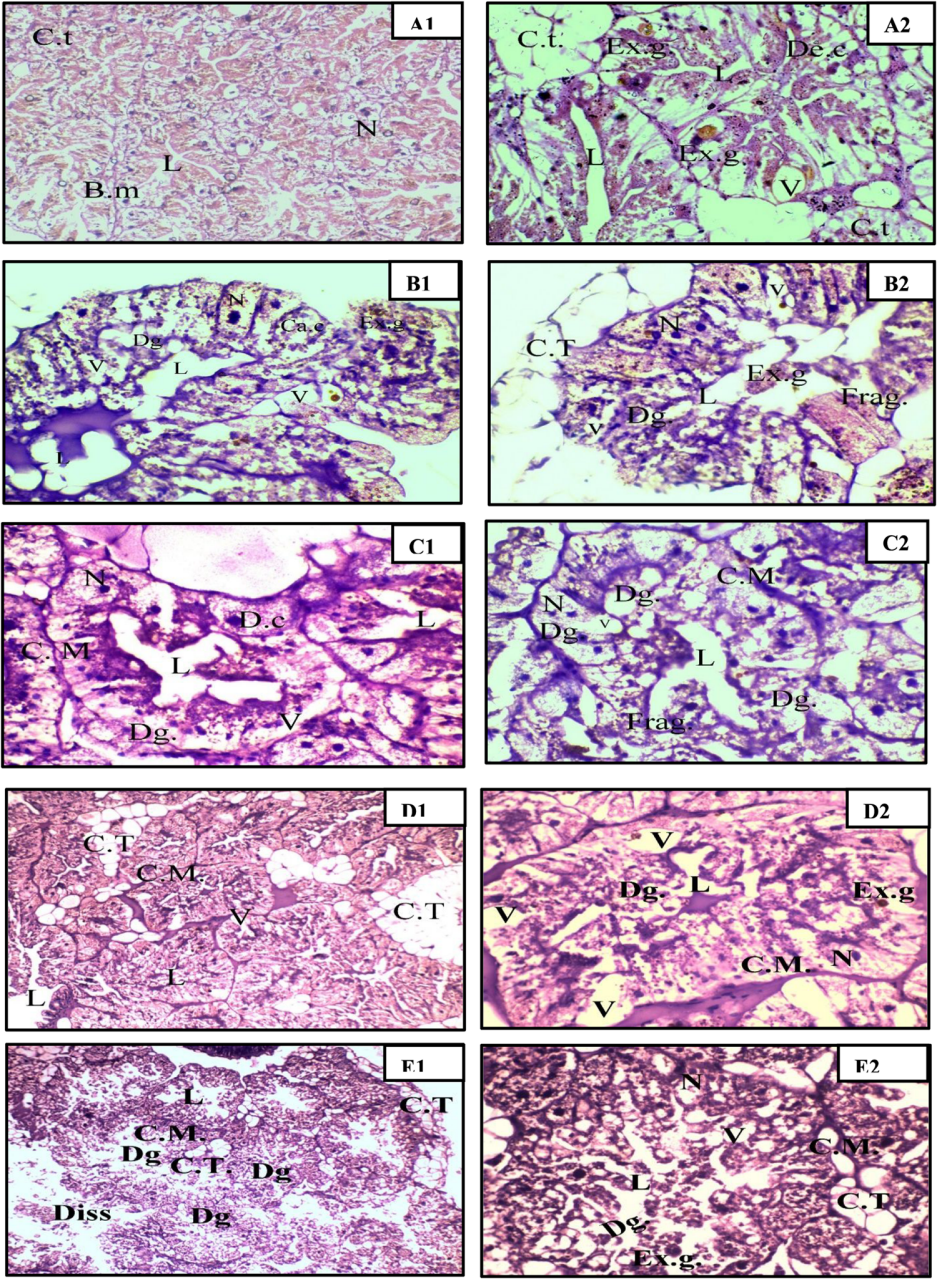
Histopathological examination of the digestive gland of land snails. (A1, A2) Control group showing intact glandular architecture at X100 and X400 magnifications, respectively. (B1, B2) Snails treated with compound **17**, exhibiting cellular degeneration, vacuolation, and lumen enlargement (X400). (**C1**, **C2**) Snails treated with compound **11**, showing nuclear displacement and fragmentation of the basement membrane (X400). (D1, D2) Snails treated with compound **18**, displaying acini shrinkage and increased vacuolation at X100, X400, respectively. (E1, E2) Snails treated with compound **16**, revealing extensive tissue degeneration, membrane fusion, and loss of structural integrity at X100, X400. Abbreviations: CT (connective tissue), BM (basement membrane), L (lumen), Ca C (calcium cell), Dg C (digestive cell), N (nucleus), Ex. g (excretory granule), V (vacuole), Ex C (excretory cell), Diss (dissociation), Frag. (fragmentation).

A thorough examination of the gland acini treated with compound **16** revealed extensive cellular degeneration (Figure 3E1,E2). All cell types lose their membranes and fuse, leading to release the intracellular contents into the lumen, which exacerbates degeneration and promotes increased vacuole formation. Most nuclei are absent, and excretory granules become fragmented and migrated into the lumen, resulting in a loss of its normal structural integrity. Furthermore, the connective tissue surrounding the acini is compromised, contributing to a significant disruption of the gland’s overall architecture and its functional roles in digestion and toxin clearance. The effects of compound **16** closely resemble those induced by methomyl, as reported in previous studies. The treatment of the land snail with methomyl resulted in pronounced degeneration and rupture of both digestive and excretory cells, accompanied by extensive vacuolization and hemolymph infiltration.^12^

### 3.3 Homology Modeling

While our research is primarily focused on the development of potent molluscicides against snails, the protein data bank (www.rcsb.org) lacks a GABAA crystal structure from this particular species. The nearest complete structure comes from a sample (3RHW). At the same time, the primary sequence of a ((Linnaeus, 1758), great pond snail) GABAA GluCl zeta subunit is available on UniProt databases. First, we elected to use homology modeling between the 3RHW roundworm structure and the pond snail zeta sequence to generate a chimeric model comprising of 4 units, and 1 unit. At the same time, we studied the standard 3RHW model. As such, all of our computational studies were run in parallel against 3RHW and the 3RHW-zeta chimera (Figure 4A–D).

**Figure 4:**
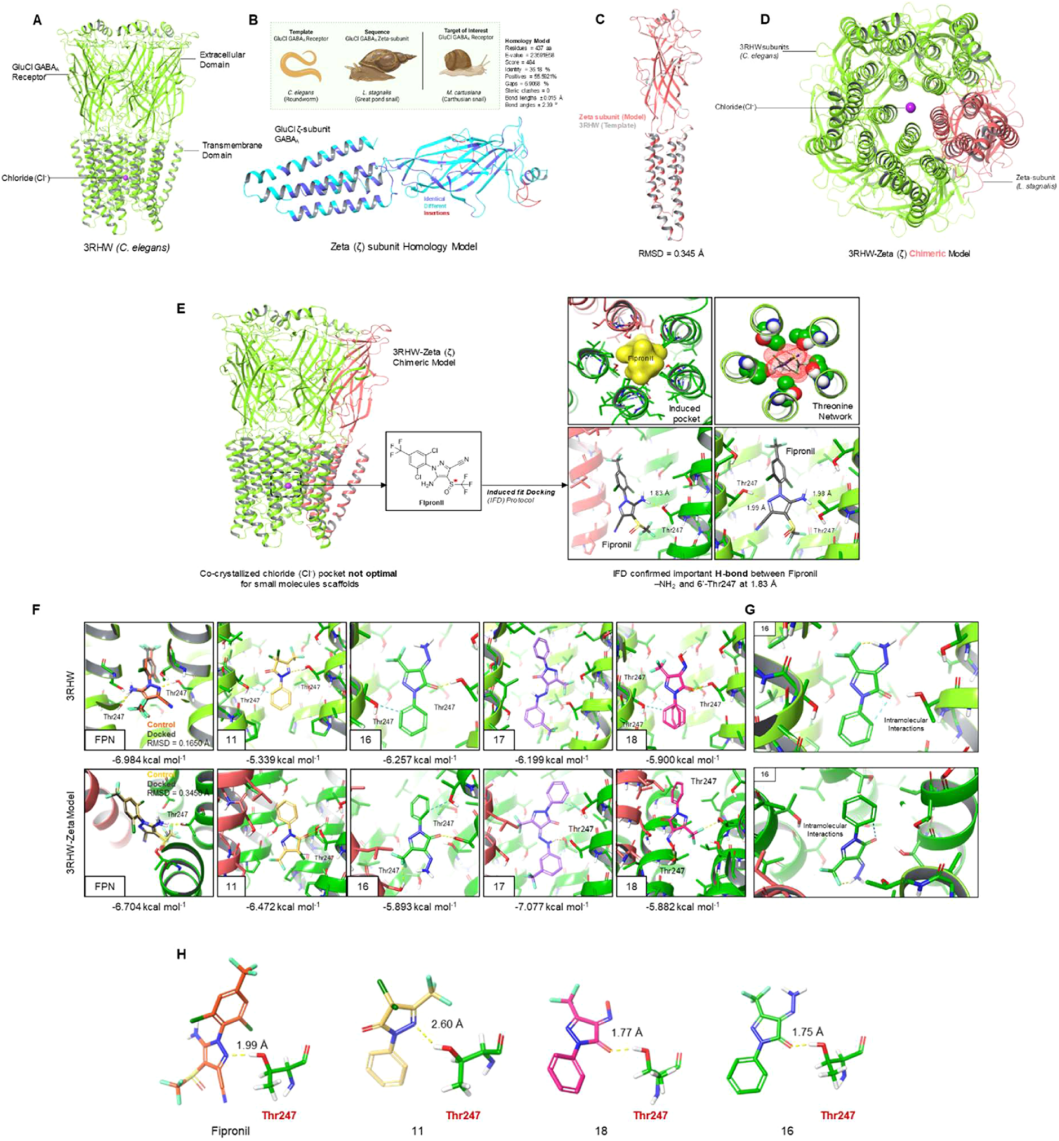
(A). Ribbon diagram of the glutamate-chloride (GluCl) GABAA receptor (3HRW, ) highlighting the cocrystallized chloride ion (Cl^–^), transmembrane domain, and extracellular domain. (B). Homology modeling strategy using the structure as a template using the closely related GABAA zeta-subunit (as a model for M. cartusiana). The resulting model is shown in blue (identical), sky blue (different), and red (insertions). Model quality is highlighted in the figure using several quantities: Score, *e*-value, identity, positives, gaps and deviations in bond lengths/angles. (C). Structural superposition between the 3RHW GABAA subunit () and the GluCl GABAA zeta-subunit () demonstrating strong alignment (RMSD = 0.345 Å). (D). Structure of the 3HRW-zeta subunit chimeric model generated with 4 3HRW subunits and 1 zeta subunit (homology). (E). Induced fit docking (IFD) protocol on the 3HRW-zeta chimeric model using Fipronil as a literature standard and general trifluoromethylpyrazole scaffold. IFD complexes in both 3RHW and 3RHW-zeta models returned hydrogen bond interactions between the pyrazole amino group and neighboring Thr247 residue (1.83, 1.98 Å), as suggested in previous research. The threonine network of the GluCl GABAA receptor is also highlighted. (F). Receptor–ligand docking results for trifluoromethylpyrazolone compounds 11, 16, 17, 18 along with Fipronil (as a positive control). Maestro/Glide extra precision (XP) docking protocols were used to derive free energies of binding (docking scores, kcal mol^–1^). (G). Compound 16 docked in the GluCl GABAA receptor of both 3HRW, and 3HRW-zeta models, highlighting key intramolecular interactions which are predicted to stabilize the top binding pose (lowering entropic penalty of binding). (H). Hydrogen-bond analysis of Fipronil and compounds 11, 16, and 18 focusing on H-bond bond length to estimate bond strength (very strong = 1.50–2.00 Å, strong = 2.00–2.50 Å, moderate = 2.50–3.20 Å, weak = 3.30–4.50 Å).

After extracting the target sequence of the zeta-subunit (Q08861, 437 residues, 50,013 Da), a knowledge-based BLAST homology workflow was performed, maintaining the 3HRW GABAA roundworm structure as a template. After posthomology sequence alignment, a chimeric model was generated (*E*-value = 2.36 × 10–58, score = 484, identity = 36.18%, positives = 55.59%, gaps = 6.91%). Steric clashes were minimized, and high-energy amino acid conformations were relaxed to create a zeta-subunit model with strong alignment to the 3RHW template (RMSD < 1.00 Å). Homology model generation was also confirmed via protein quality reports (steric clashes = 0, bond length deviations = 0.015 Å, angle deviations = 2.39°) and Ramachandran plots (∼98% of residues in allowed regions).

#### 3.3.1 Induced Fit Docking

Given the lack of a Fipronil-GABAA cocrystal, the exact binding site of FPN on GABAA has been in question for several decades.^48–54^ Multiple reports suggest that FPN physically blocks the chloride pore, while other studies suggest this molecule acts as a noncompetitive inhibitor of allosteric sites within the GABAA channel.^16,55^ The first hypothesis represents the current literature consensus, and is indirectly supported by various biochemical, electrochemical, and biophysical studies.^48,49^ Following this, we turned our attention to the chloride pore (Figure 4E).

Both the 3RHW (roundworm) GABAA GluCl and our zeta-chimera contain a chloride ion in the channel pore. As such, this pore size (i.e., binding pocket) is nonoptimal and too restricted for the screening of trifluoromethylpyrazolone scaffolds (which are much larger). To solve this, we performed an induced fit docking (IFD) experiment. While most docking studies assume a rigid receptor, IFD uses a reference scaffold (e.g., FPN) and a pocket of interest (e.g., Cl- pore) to allow residue side chains within a set distance be dynamic to generate an induced binding pocket, which is more suitable for the ligands of interest. Molecular docking would be performed on this new (larger) pocket. In this way, an induced-fit experiment was run on 3HRW and the zeta-subunit chimera replacing the Cl- ion with FPN and a 5 Å side chain VDW scaling of 0.70. From the results, we selected a receptor–ligand complex which best previous literature, with the trifluoromethyl motif facing the extracellular domain and the pyrazole amino group forming a hydrogen bond with the 6′ Thr247 (1.83–1.98 Å), which is known to be a key interaction for phenylpyrazole GABAA binding.^16^ Favorable IFD scores were found for the 3RHW and 3RHW-zeta models, at −3226.42 and −3355.04 kcal/mol, respectively (Figure 4E). Note, FPN contains a chiral center at the sulfoxide sulfur atom. While several research articles have found different enantiomers to be slightly better in different species, this molecule is marketed as a racemate. As such, we used R-fipronil as a model for our studies as we noticed no statistically significant difference when compared to the S-enantiomer.

#### 3.3.2 Receptor–Ligand Docking

Using a more appropriate induced protein structure, we then performed a series of docking experiments on both 3RHW and 3RHW-zeta models to determine free energy docking scores (kcal/mol) for the inhibitors; *gem*-dichloro (**11**), hydrazone (**16**), phenylhydrazone (**17**), and oxime (**18**). This study involves ligand preparation, protein preparation, receptor-grid generation, ligand docking, and postdocking pose analysis. The *N*-methyl carbamate methomyl and R-fipronil were selected as positive controls.

Ligands were prepared with a standard LigPrep workflow using Maestro glide/epik modules. This involved ionization at a target pH of 7.4 ± 2.0, desalting, tautomer generation, retained chirality and energy minimization into three-dimensional low energy conformers. Both the phenylhydrazone (**17**) and the oxime (**18**) generated ionized species in this step. While the IFD study already involved a protein preparation step for both the 3HRW and 3HRW-zeta models, these results were revalidated at this stage through checking hydrogen additions, bond orders, missing side chains, steric clashes, missing atoms, and the optimization of hydrogen-bond networks. We then generated a receptor-grid for both models, which specifies a cubic volume for the ligands to survey during docking. The grid was defined around the R-fipronil ligand (out of the IFD study) and was sized at a standard 10 Å.^50^ Given the importance of the GABAA Thr networks for phenylpyrazole molecular recognition, all Thr residues were allowed to rotate, and no other constraints were set. First, a control experiment was run to redock FPN into the same pocket and ensure correct experiment parametrization. In both the 3HRW and zeta-subunit chimera model, the top 3 binding poses of FPN were strongly aligned with the reference model, with low RMSD ranges of (0.00–0.3450 Å) and (0.1065–0.1651 Å), respectively, while docking scores of the top pose were found to be −6.796 and −7.080 kcal/mol, respectively. The Thr247 H-bond was conserved in all poses, either at the amino group, or the pyrazolone nitrogen atom. In this way, docking experiments were run for our 4 inhibitors: **11**, **16**, **17**, and **18** using standard precision (SP) glide workflows and the results are summarized in Figure 4F,G. Starting with the standard 3RHW model (), the halogenated **11** found a docking score of −5.339 kcal/mol with a H-bond to Thr247, along with a weaker aromatic H-bond with the benzene hydrogen atom. Hydrazone **16** had a docking score of −6.257 kcal/mol, with top binding poses having H-bonds between the pyrazolone carbonyl and Thr247 as well as between the free amino group and Thr251. In fact, **16** showed the highest docking score, which is in line with the *in vivo* data against (LC_50_ = 0.58 mg/mL). While compound **17** also returned a strong docking score of −6.199 kcal/mol, interestingly, it lacked any H-bonds (including to Thr247) (Figure 4F,G).

This suggests the binding event is entropic and most likely driven by hydrophobic contacts. Despite having a similar docking score to the much smaller **16**, it is also worth considering the ligand efficiency (LE) of these molecules, which takes into account the free energy of binding (i.e., docking score) as a function of size (i.e., non-hydrogen atoms) (LE = Δ*G*/HA). An LE treatment of both **16** and **17** would clearly highlight that hydrazone **16** is a more efficient ligand. Oxime **18** was found to have a reasonable score of −5.900 kcal/mol with H-bonds to Thr247. Interestingly, this trend was not entirely maintained in the 3RHW-zeta chimeric model, with scores of **11** (−6.920 kcal/mol), **16** (−5.893 kcal/mol), **17** (−7.007 kcal/mol), and **18** (−5.882 kca/mol). This is likely due to the zeta-subunit homology model positioning the critical Thr247 in a slightly different trajectory. At the same time, it was interesting to note that in the 3RHW-zeta model dockings, in almost all cases, the ligands were inverted with the phenyl group facing the extracellular domain. Positive control fipronil returned a strong docking score of −8.057 kcal/mol, which was expected as the grid was generated based on this molecule, while methomyl observed a weak value of −3.779 kcal/mol. Since methomyl inactivates the protein through a covalent mechanism, the result of this docking is likely not entirely representative of its free energy score.

Given that it better matches the *in vivo* data against , we elected to focus on the 3RHW GABAA model (roundworm). As the top binder, the results of hydrazone **16** (−6.257 kcal/mol) can be explained with three observations. First, this molecule was able to make multiple H-bond contacts, to Thr247 and Thr251. Upon closer inspection, compound **16** was also found to possess intramolecular bonds between the free amino group and the trifluoromethyl, as well as between the pyrazolone carbonyl and the aromatic hydrogens in the neighboring phenyl ring. These interactions likely lock the molecule in a stable binding pose, lowering the entropic binding penalty (ΔSbind) and improve the overall free energy. Third, the hydrogen bond to Thr247 is also relatively stronger when compared to the other ligands (as a function of distance).

#### 3.3.3 Hydrogen Bond Analyses

Several of our inhibitors (**11**, **16**, **18**) were found to form a hydrogen bond to Thr274, however each molecule returned a different docking score. As a whole, H-bonds are widely known to be highly dependent on distance, and angle.^56^ In fact, based on the donor–acceptor distances, a H-bond can be categorized as almost very strong (1.5–2.0 Å), strong (2.0–2.5 Å), moderate (2.5–3.2 Å), and weak (3.3–4.5 Å). In this way we measured the H-bond distances between **11**, **16**, **18** and the Thr247 hydroxyl group (Figure 4H). As expected, (and in line with our docking score trends), hydrazone **16** was found to have the strongest H-bond with a distance of 1.75 Å, followed by oxime **18** at 1.77 Å and dichloro compound **11** at 2.60 Å. Positive control fipronil had a value of 1.99 Å. This result supports the *in vivo* LC_50_ data around **16** with a good docking score of −6.257 kcal/mol and a very strong H-bond to Thr247 at 1.75 Å.

In conclusion, we have identified a series of 4-substituted 5-trifluoromethylpheny-pyrazolones with potent molluscicidal activity against for sustainable agriculture pest control. The chemical nature at the C4 substituent [C^4^-*gem*-Cl_2_ (comp. **11**, LC_50_ = 1.93 mg/mL), C^4^=NNH_2_ (comp. **16**, LC_50_ = 0.58 mg/mL), C^4^N = N-NHAr (comp. **17**, LC_50_ = 3.8 mg/mL), C = NOH (comp. **18**, LC_50_ = 1.11 mg/mL)] was determinant of the molluscicidal potency with the C4-hydrazono group showed surpassing potency to the current standard, Methomyl (LC_50_ = 2.28 mg/mL). Elevation of AST, ALT, and AChE levels, reduction of the carbohydrate and lipid levels, extensive damage to the digestive gland, and typical nervous system toxicity’s symptoms were observed with the treated snails. These observations suggest a dual mode of action involving gastrointestinal toxicity and GABA_A_-Glu gated chloride ion channel antagonism. Docking studies confirmed the experimental findings, providing insights into the molecular interactions between trifluoromethyl pyrazolones and GABA_A_-GluCl ion channel binding pockets. The results of this study underline the importance of trifluoromethyl-phenylpyrazolones as a promising scaffold for the development of safer and more effective molluscicides, aligning with sustainable pest control practices and global food security goals.

## Supplementary Material



## Notes

Synthetic procedures and full spectroscopic data (^1^H NMR, ^13^C NMR, and ^19^F NMR) for all compounds. Additional information includes biological assay protocols for Monacha cartusiana, biochemical and histological analysis methods, and computational docking studies, along with associated figures and experimental details (PDF)
