# Supplementary material for: Designing Trifluoromethyl Pyrazolones for Selective Molluscicidal Activity Against : Toward Sustainable Land Snail Control
Source: J Agric Food Chem. 2025 Jun 11;73(32):19907–20. doi: 10.1021/acs.jafc.4c12327 (PMC12355956; doi:10.1021/acs.jafc.4c12327)
Supplement: Supplementary file 1 [file jf4c12327_si_001.pdf]

Supporting Information

Designing Trifluoromethyl Pyrazolones for Selective Molluscicidal Activity Against *Monacha cartusiana*: Toward Sustainable Land Snail Control

Hend M. A. Maaroof[a,c], *Shaikha S. AlNeyadi*[*,b], *Yasir S. Raouf*[b], *Fatma I. El-Akhrasy*[c], *Abdalla E. A. Hassan*[*,a], *and Reham A. I. Abouelkhair*[a]

[a]*Applied Nucleic Acids Research Center & Chemistry Department, Faculty of Science, Zagazig University, Zagazig 44519, Egypt*

[b]*Department of Chemistry, College of Science, United Arab Emirates University, Al Ain 15551, United Arab Emirates*

[c]*Plant Protection Research Institute, Agricultural Research Center, Dokki, Giza 12622, Egypt*

**Corresponding Authors:*

Shaikha S. AlNeyadi – shaikha.alneyadi@uaeu.ac.ae
Abdalla E. A. Hassan – aeahassan@aol.com

S1.1. Chemistry: General

The ^{1}H-NMR and ^{13}C-APT NMR were performed on a Bruker 400 MHz (Bruker comp., MA, USA) at Applied Nucleic Acid Research Center (ANARC), Faculty of Science, Zagazig University, Egypt.

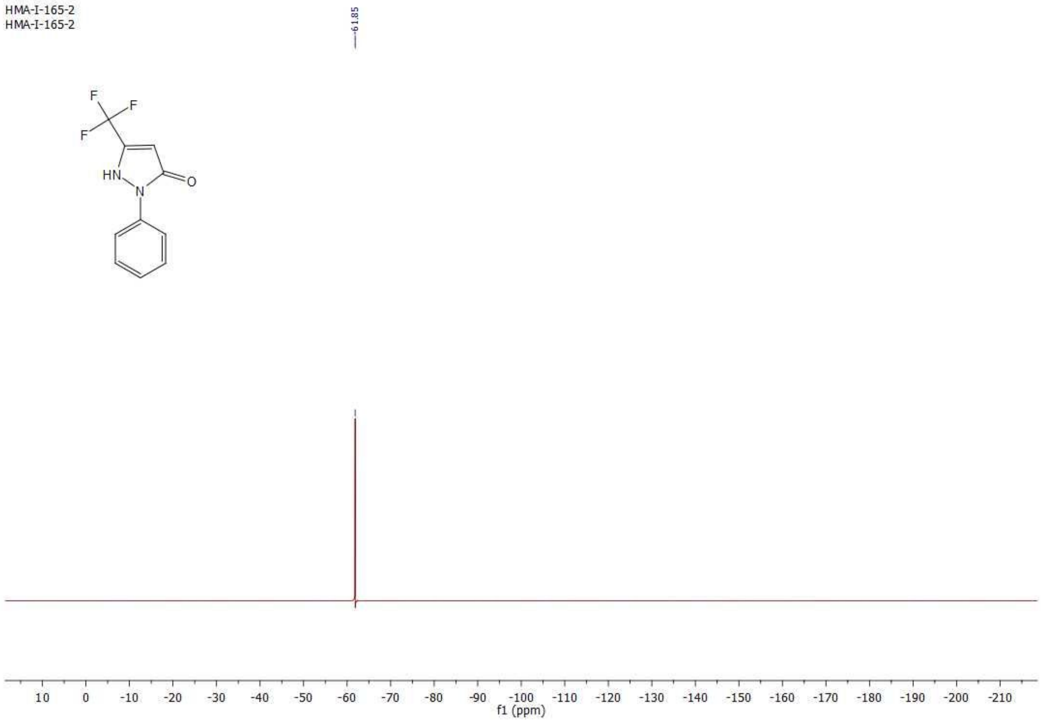

Figure S1: ^{19}F-NMR spectrum of compound **10**.

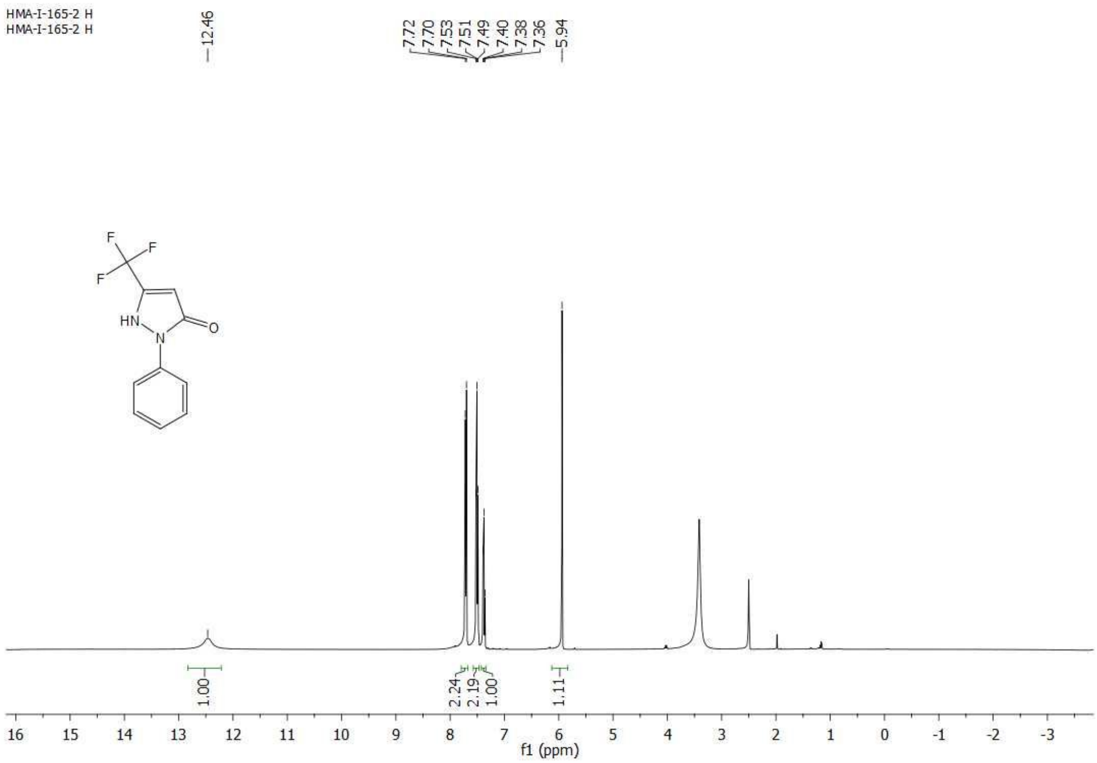

Figure S2: ^{1}H-NMR spectrum of compound **10**.

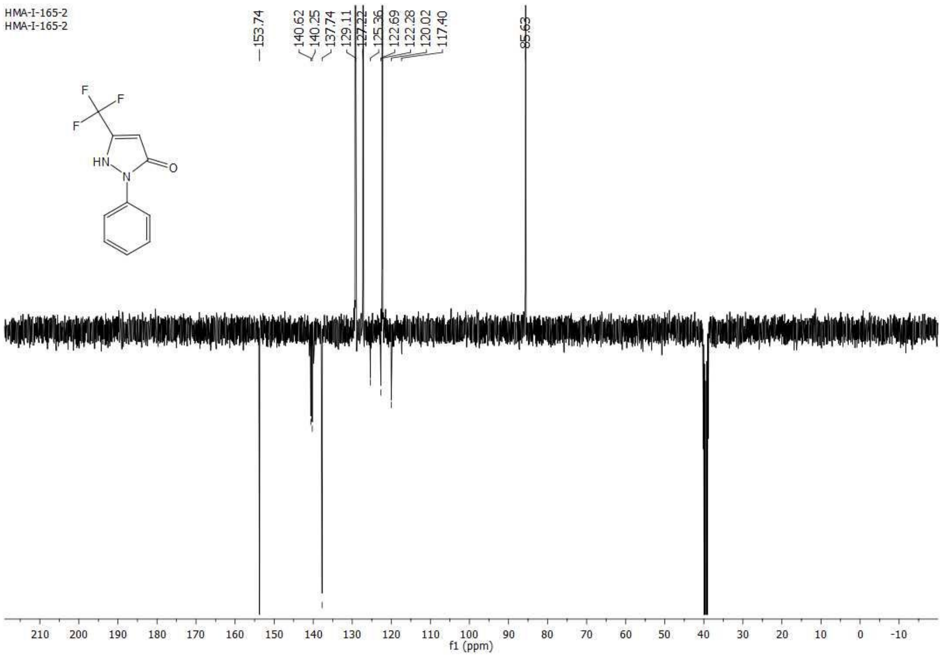

Figure S3: ^{13}C-NMR spectrum of compound **10**.

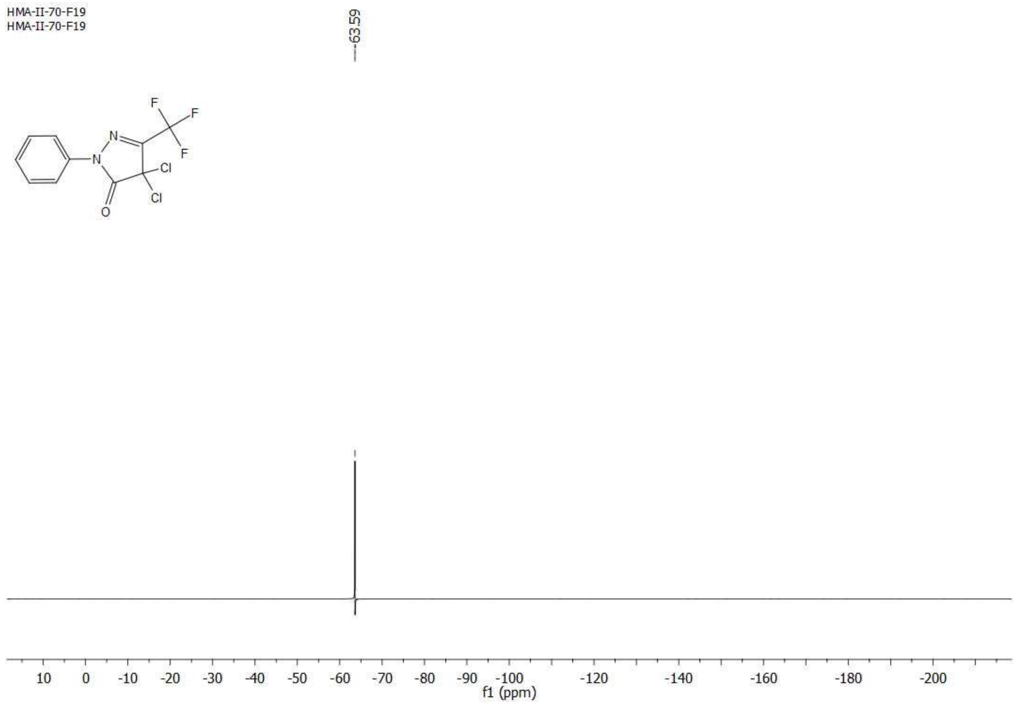

Figure S4: ^{19}F-NMR spectrum of compound **11**.

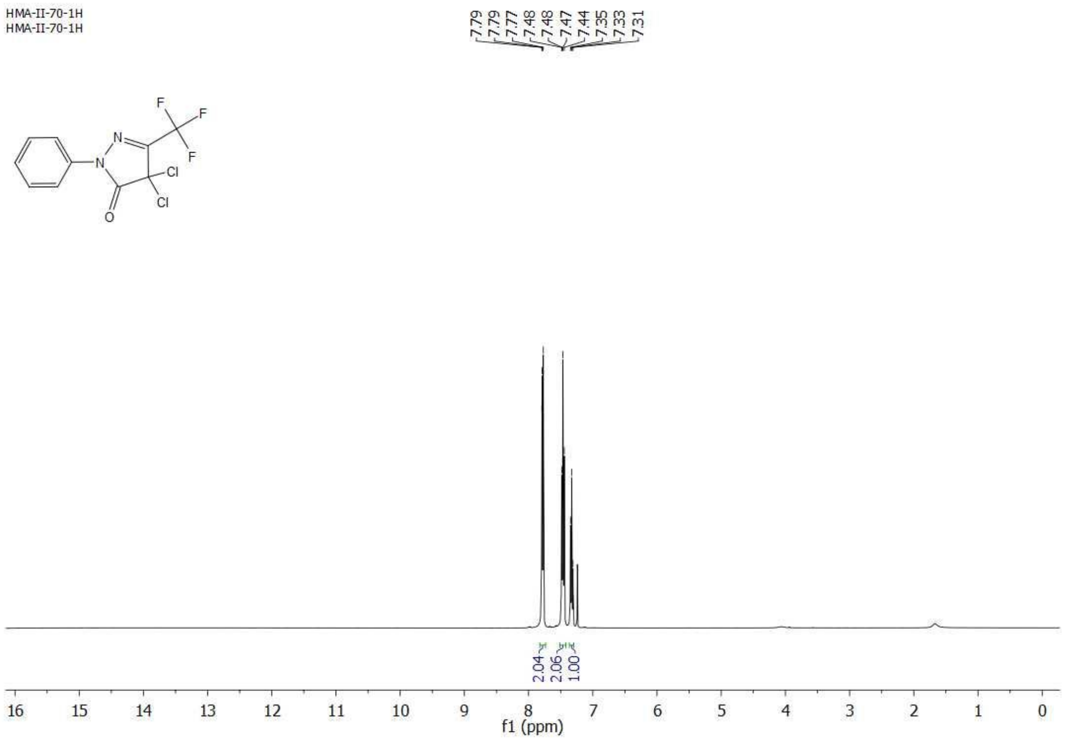

Figure S5: ^{1}H-NMR spectrum of compound **11**.

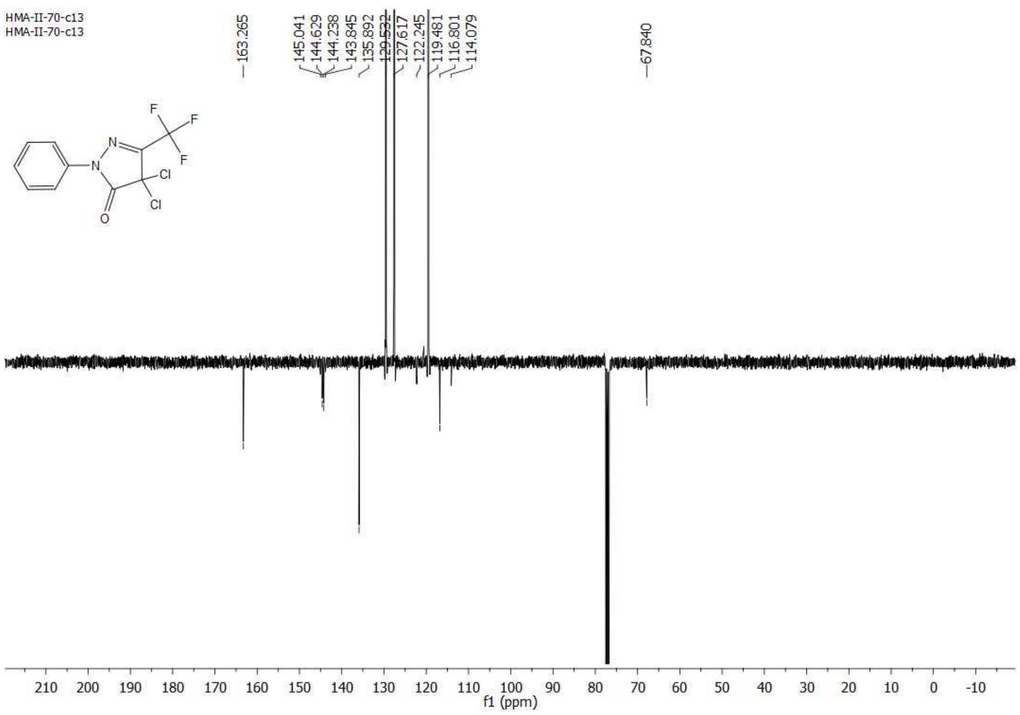

Figure S6: ^{13}C-NMR spectrum of compound **11**.

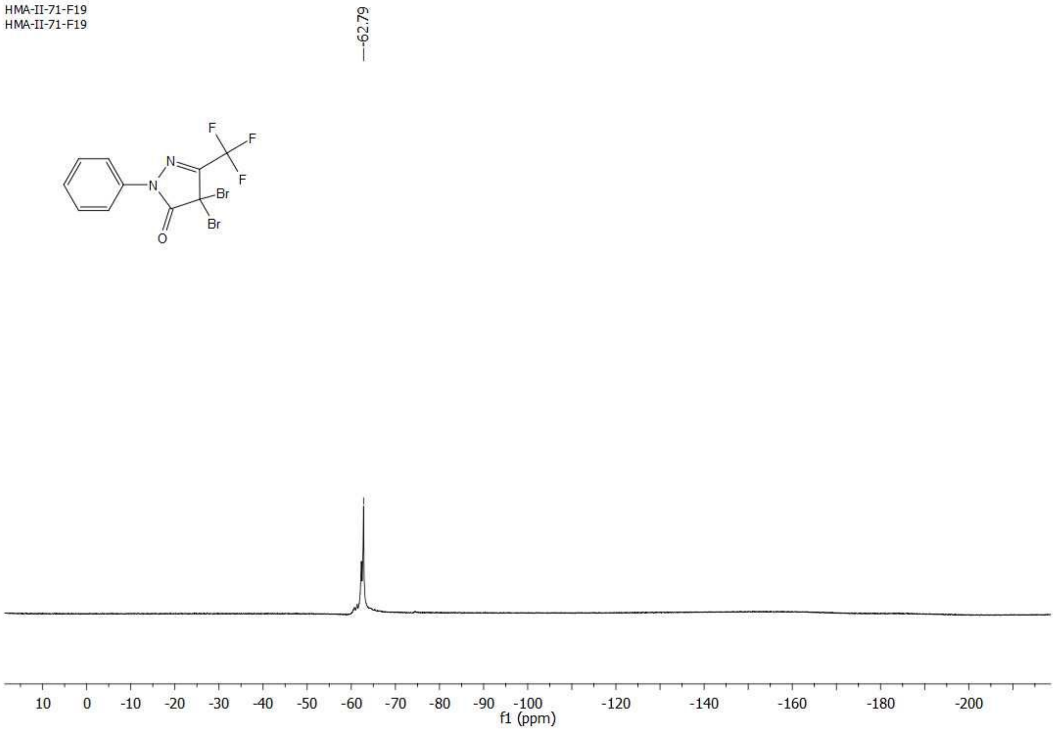

Figure S7: ^{19}F-NMR spectrum of compound **12**.

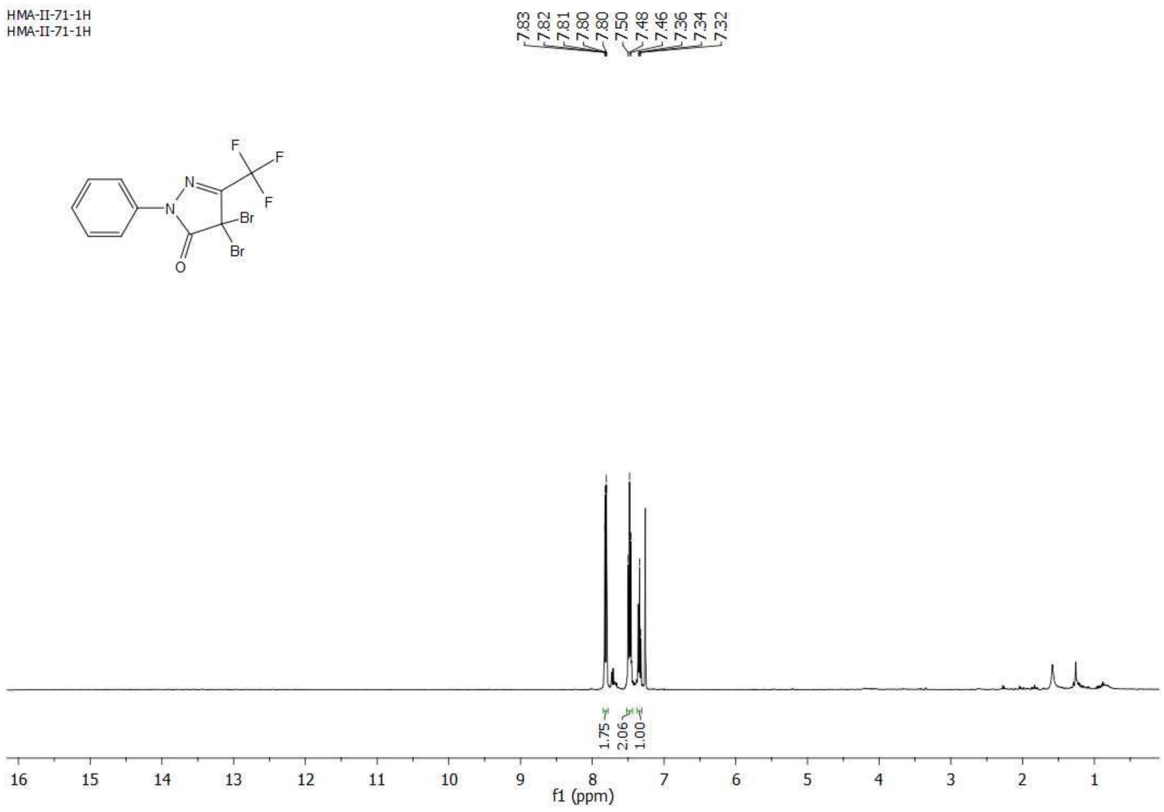

Figure S8: ^{1}H-NMR spectrum of compound **12**.

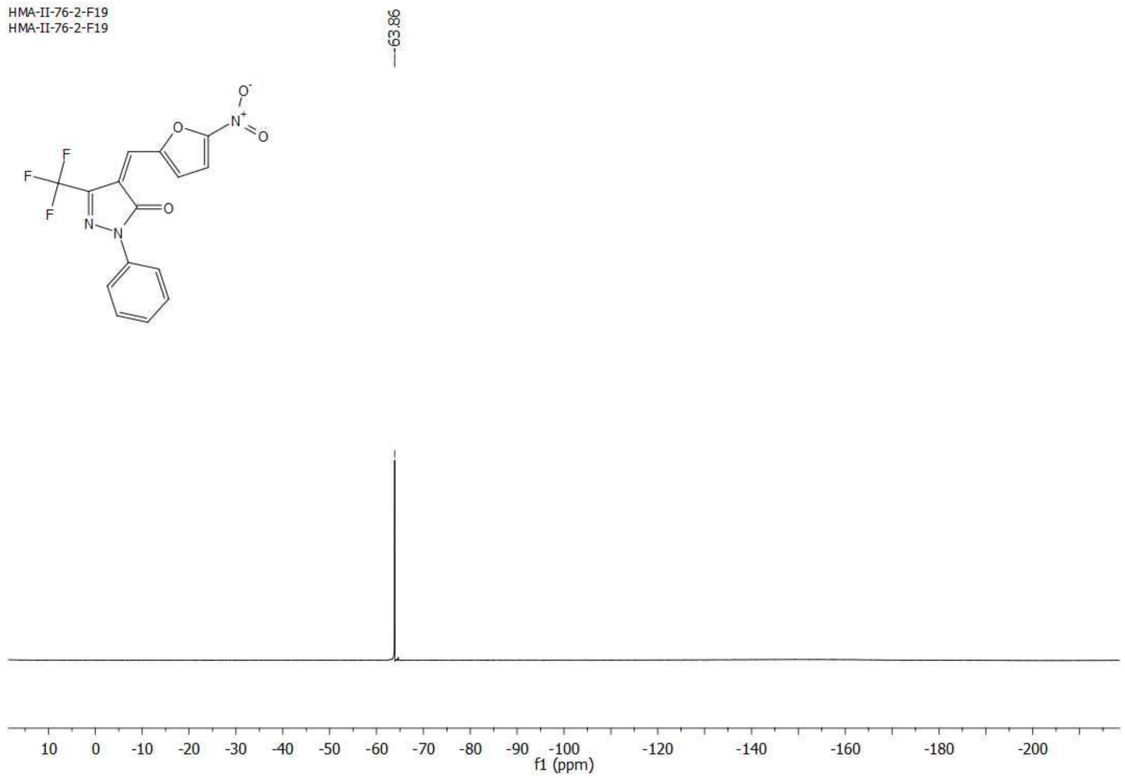

Figure S9: ^{19}F-NMR spectrum of compound **13**.

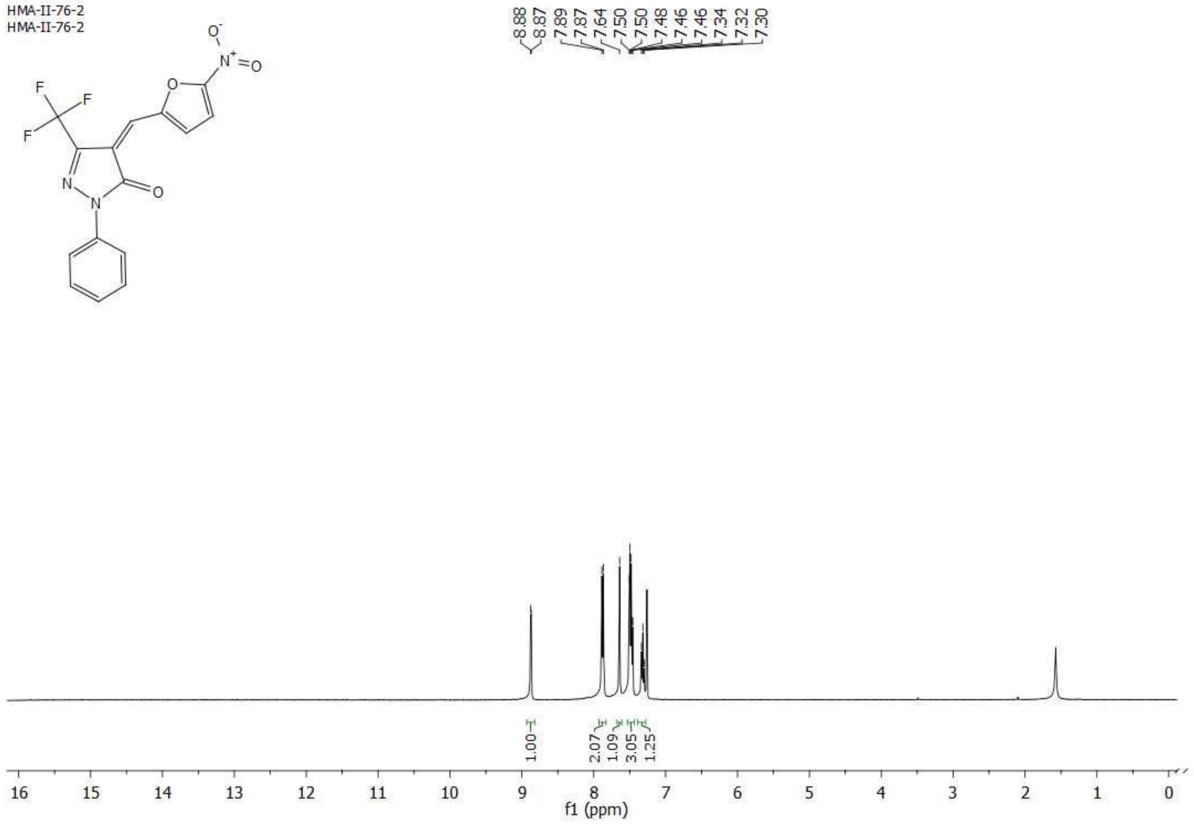

Figure S10: ^{1}H-NMR spectrum of compound **13**.

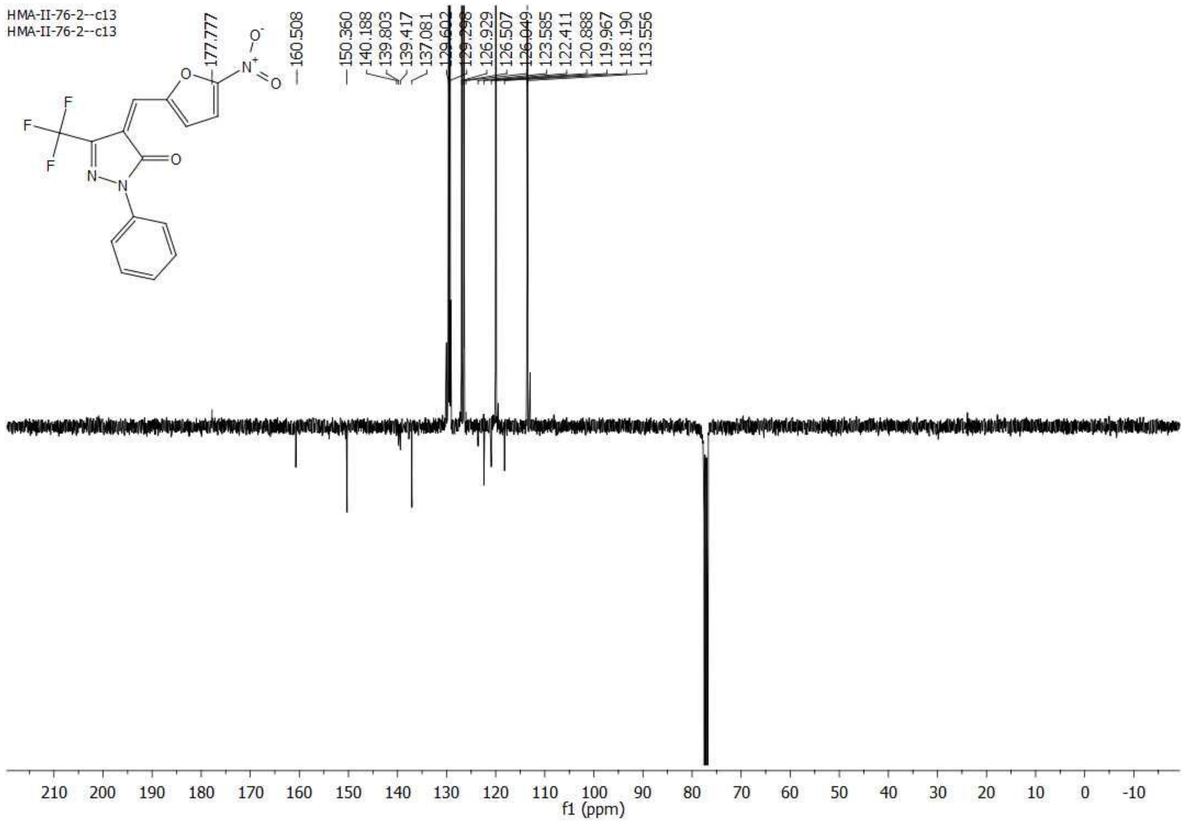

Figure S11: ^{13}C-NMR spectrum of compound **13**.

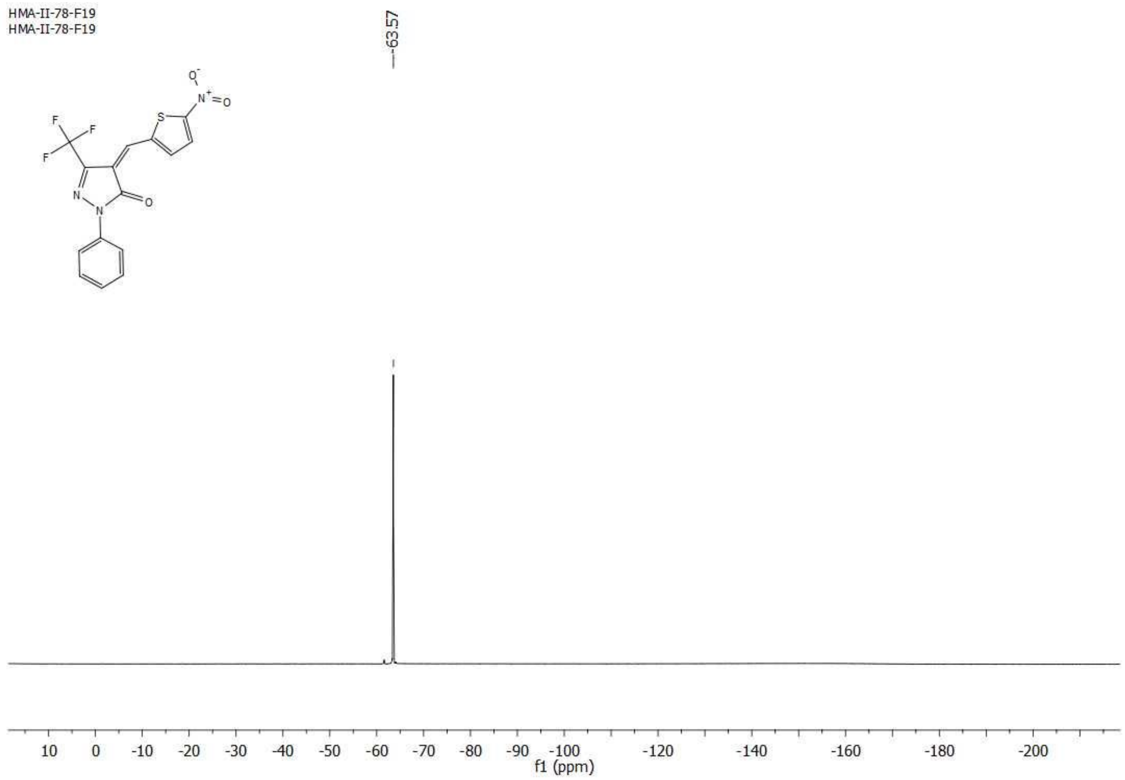

Figure S12: ^{19}F-NMR spectrum of compound **14**.

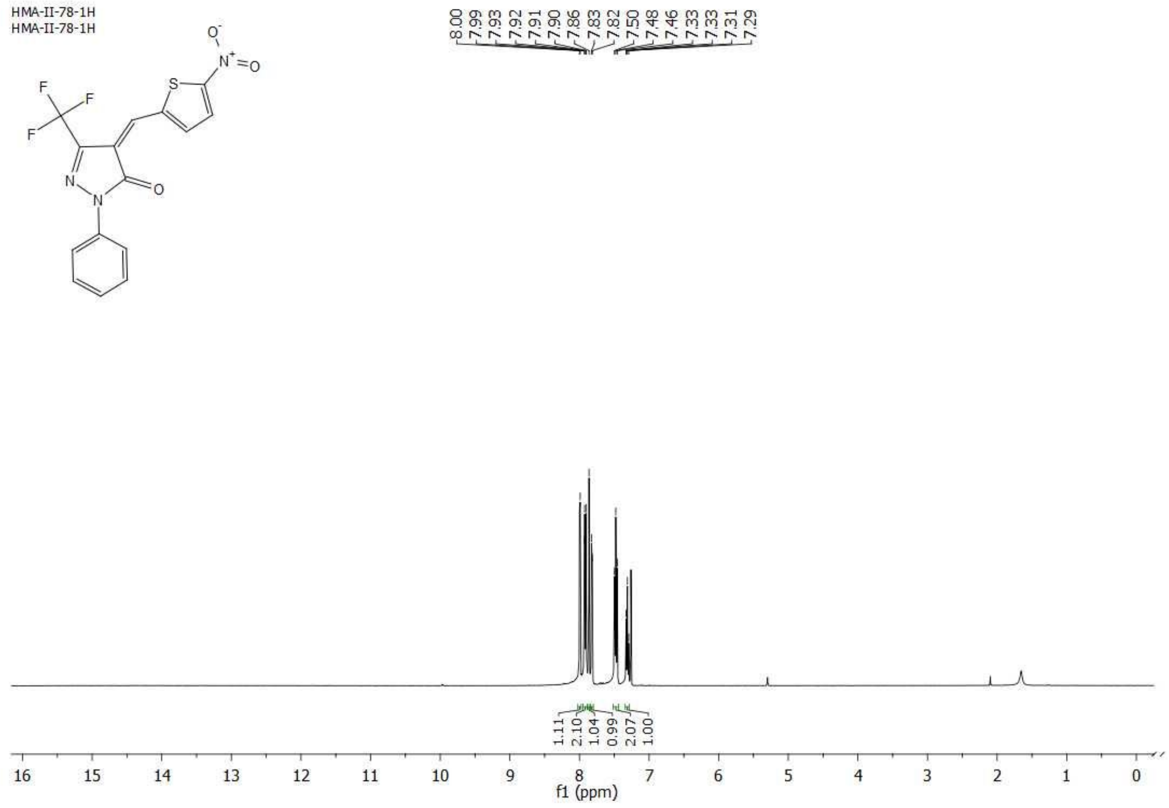

Figure S13: ^{1}H-NMR spectrum of compound **14**.

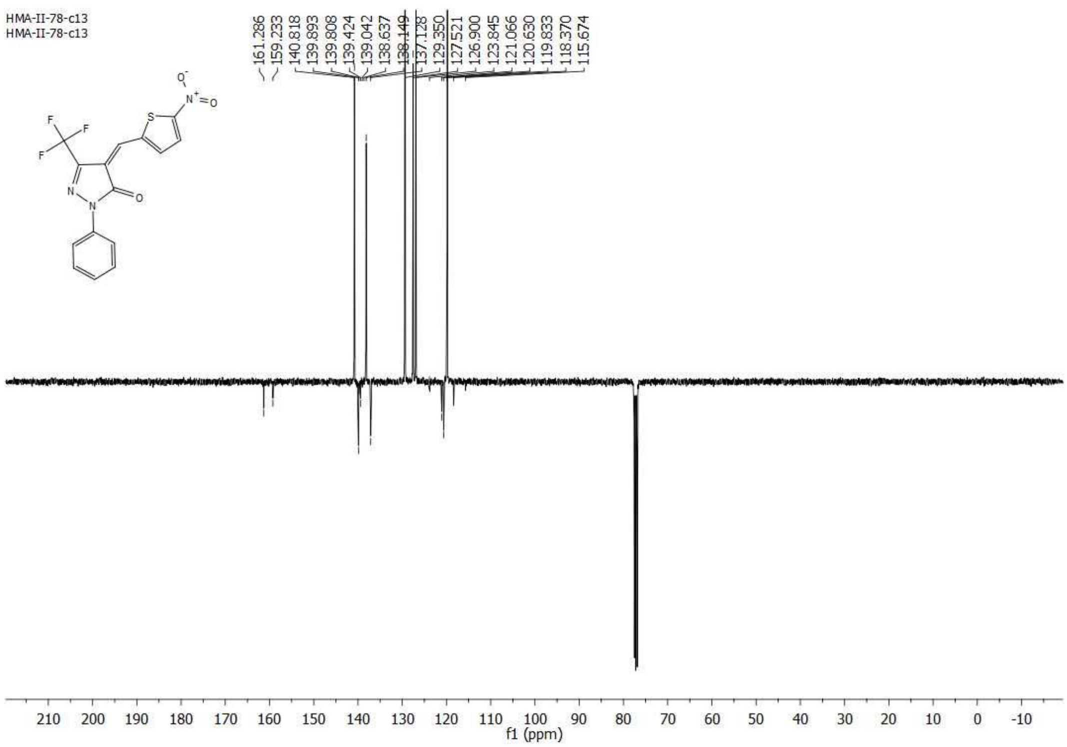

Figure S14: ^{13}C-NMR spectrum of compound **14**.

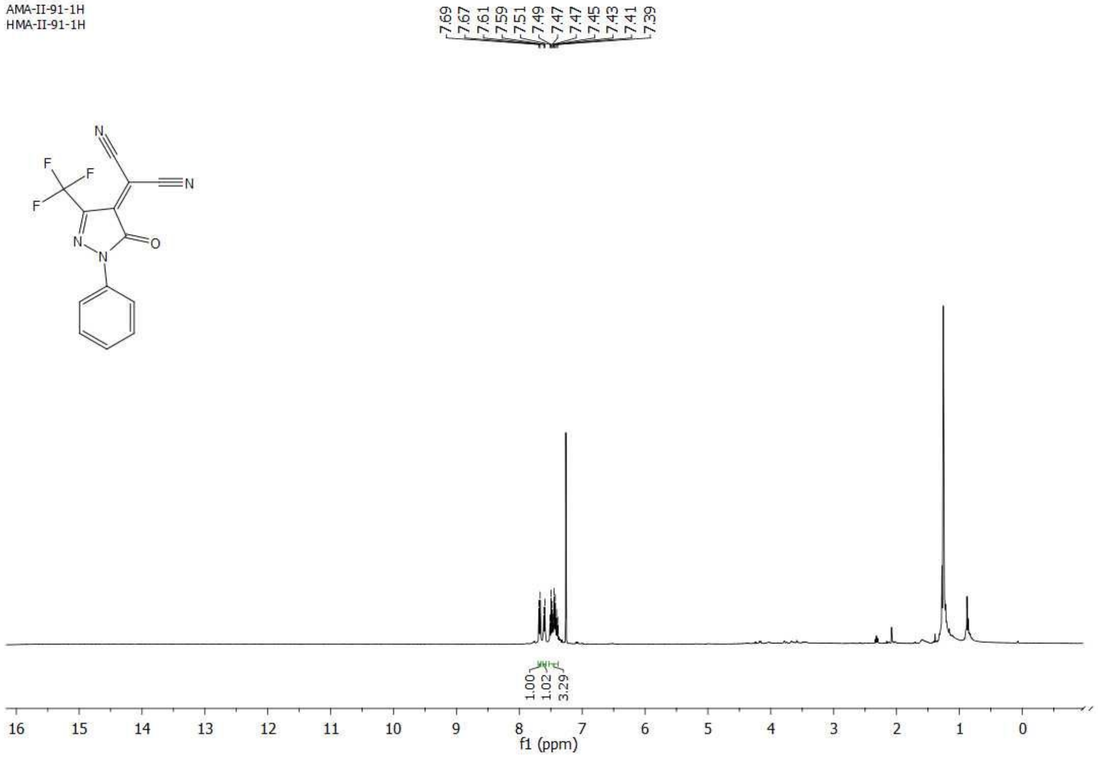

Figure S15: ^{1}H-NMR spectrum of compound **15**.

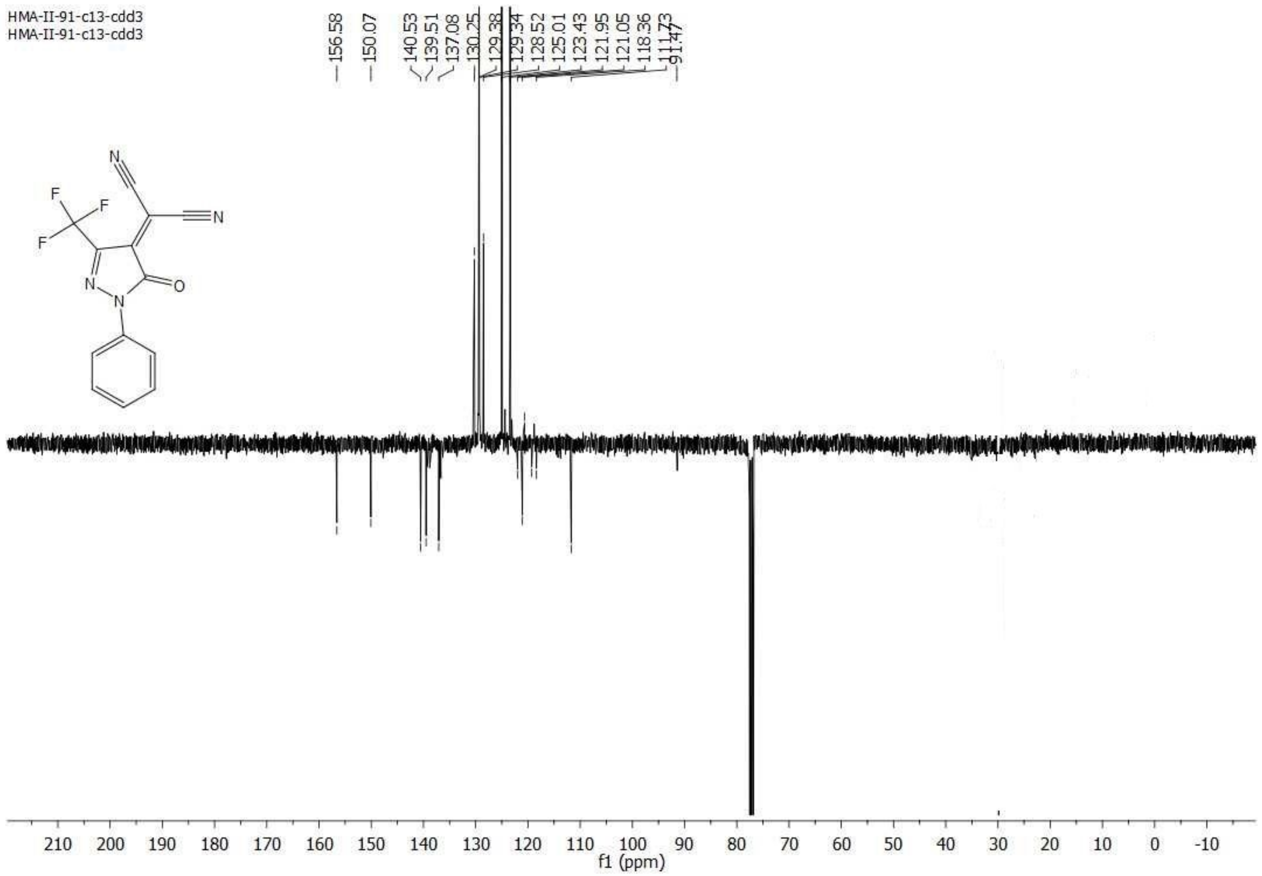

Figure S16: ^{13}C-NMR spectrum of compound **15**.

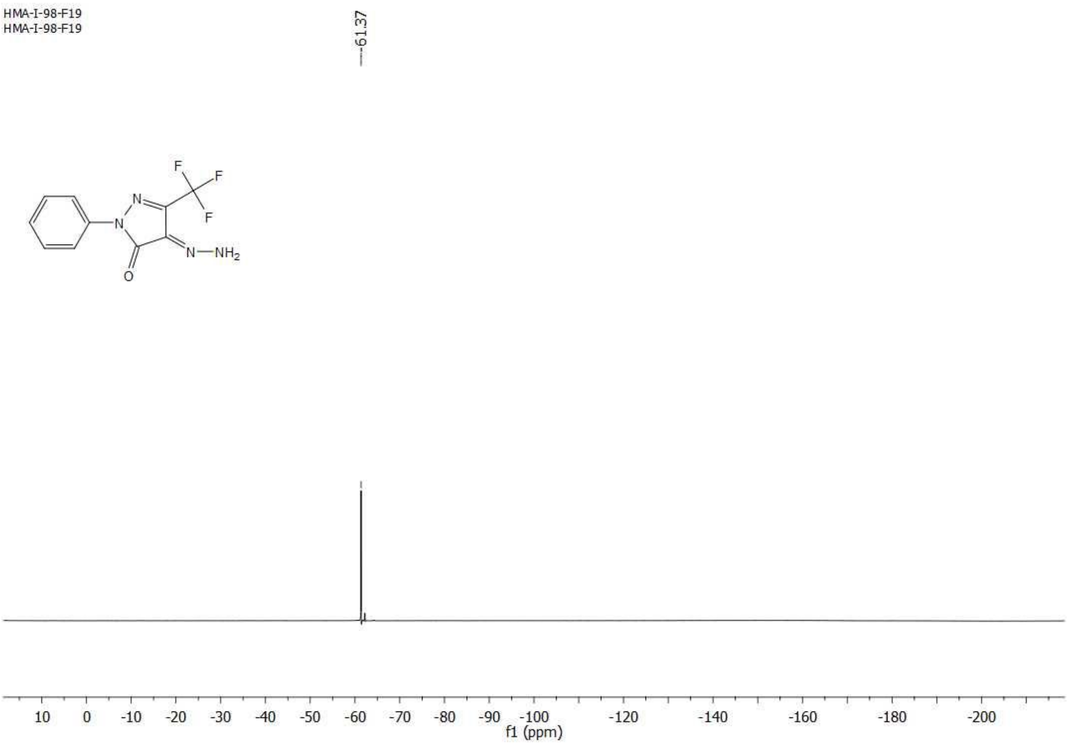

Figure S17: ^{19}F-NMR spectrum of compound **16**.

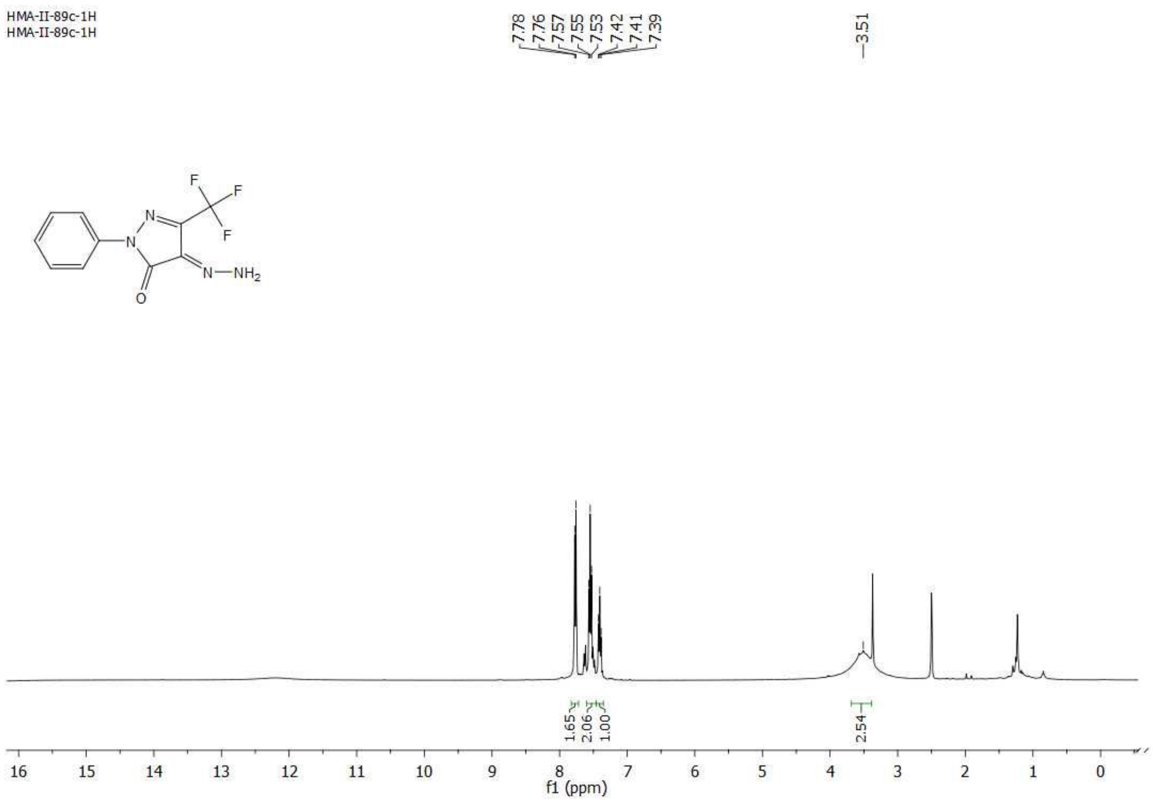

Figure S18: ^{1}H-NMR spectrum of compound **16**.

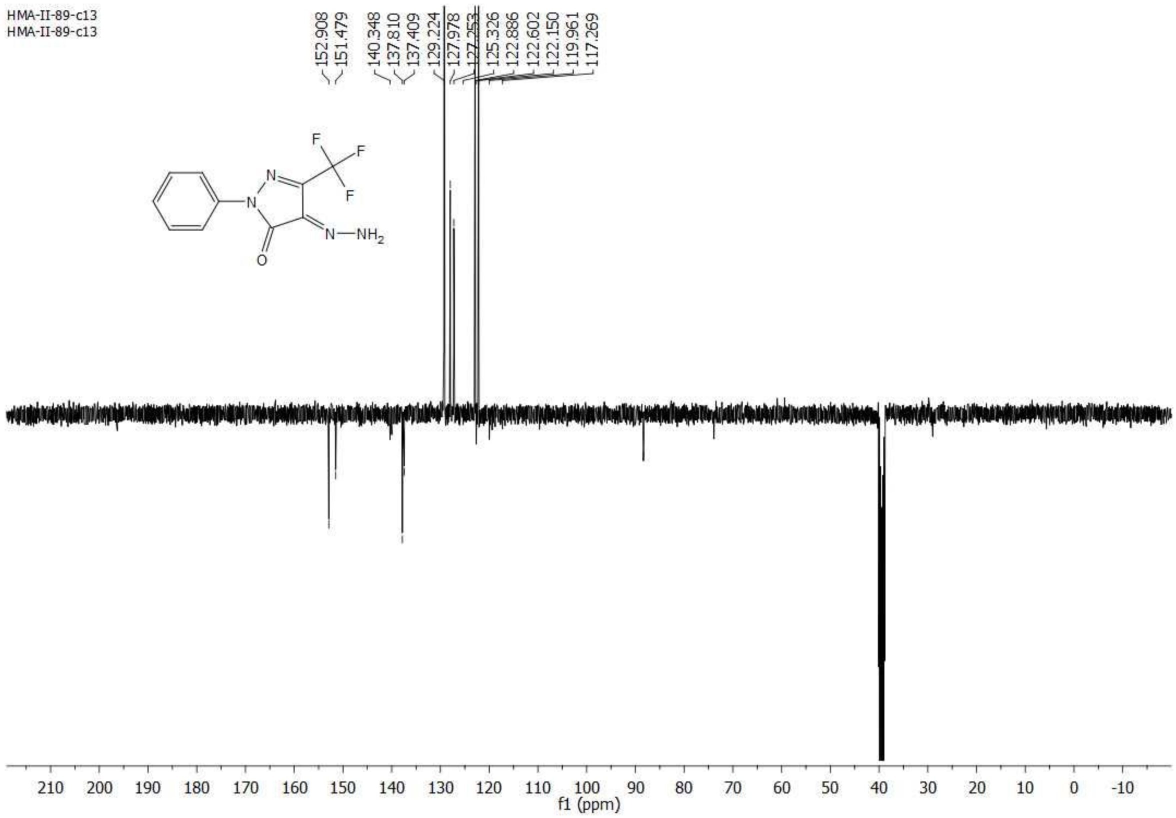

Figure S19: ^{13}C-NMR spectrum of compound **16**.

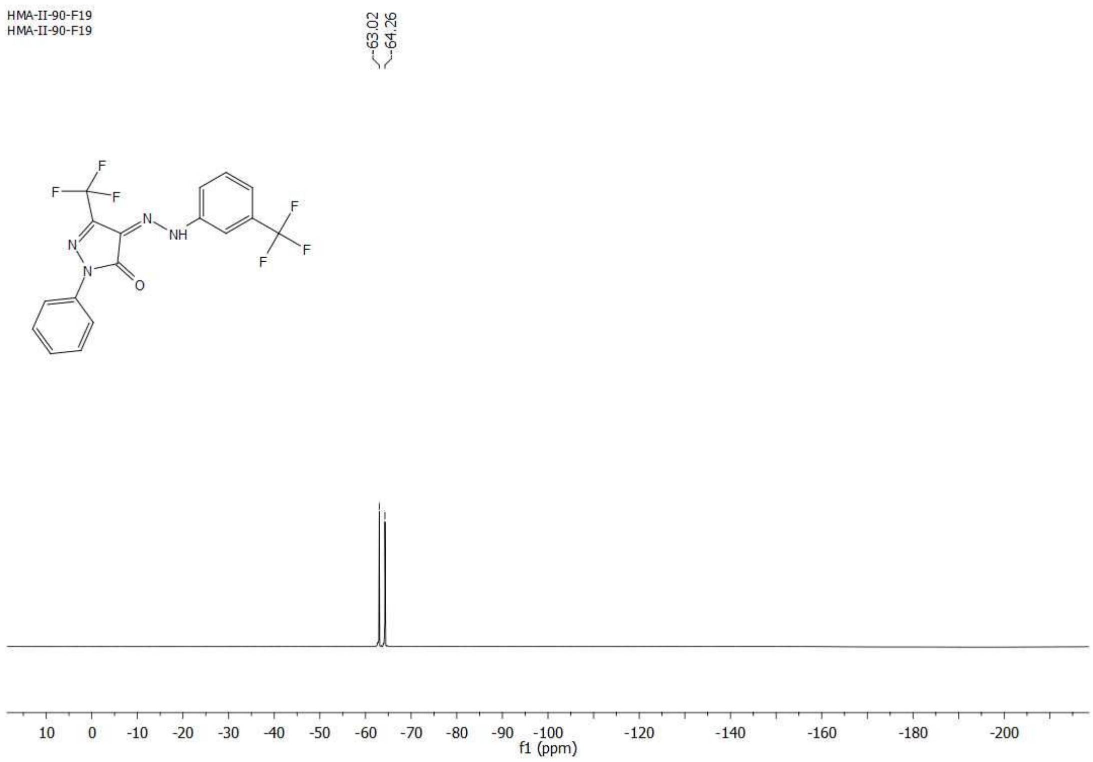

Figure S20: ^{19}F-NMR spectrum of compound **17**.

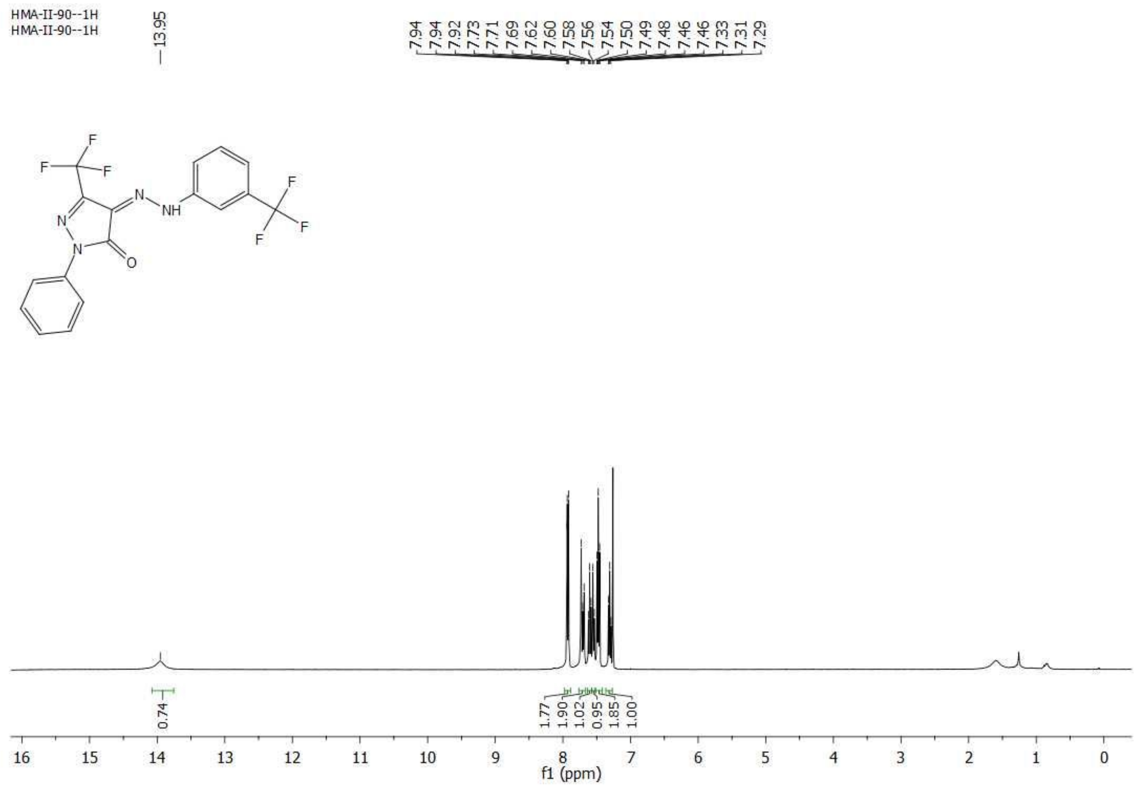

Figure S21: ^{1}H-NMR spectrum of compound **17**.

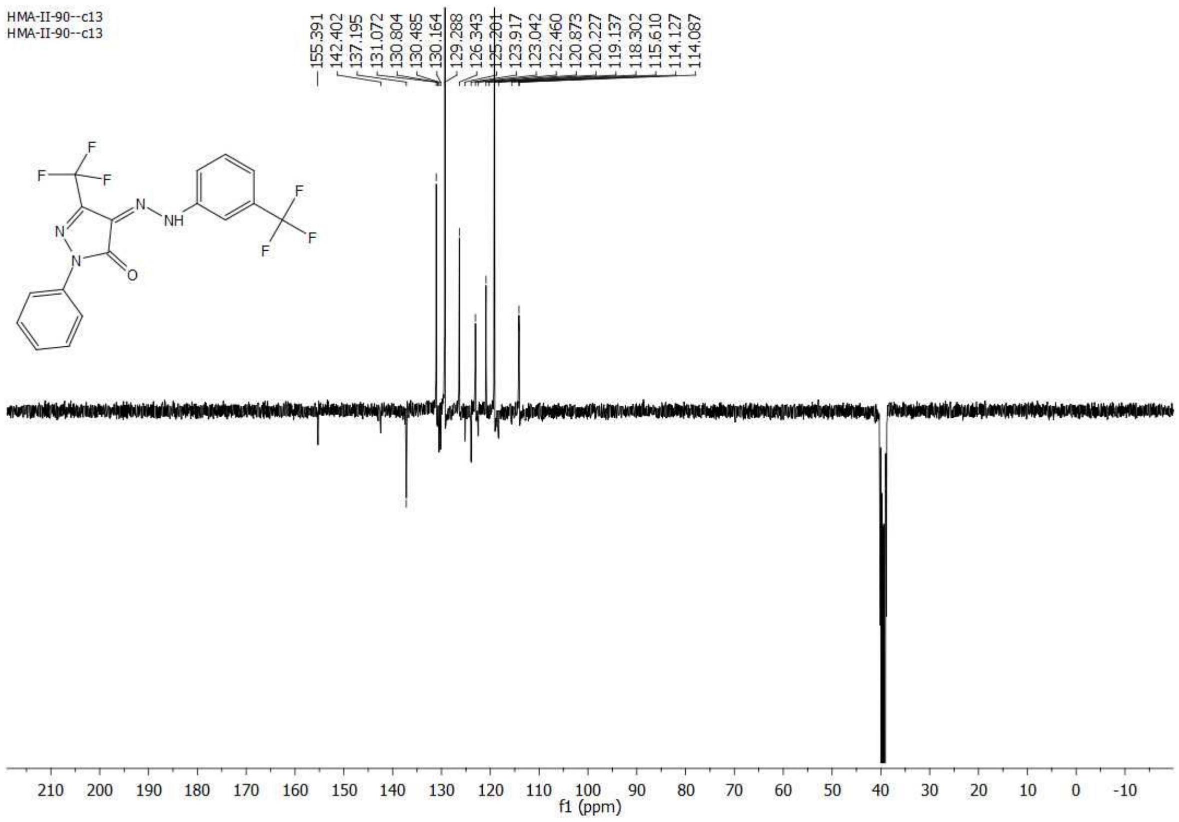

Figure S22: ^{13}C-NMR spectrum of compound **17**.

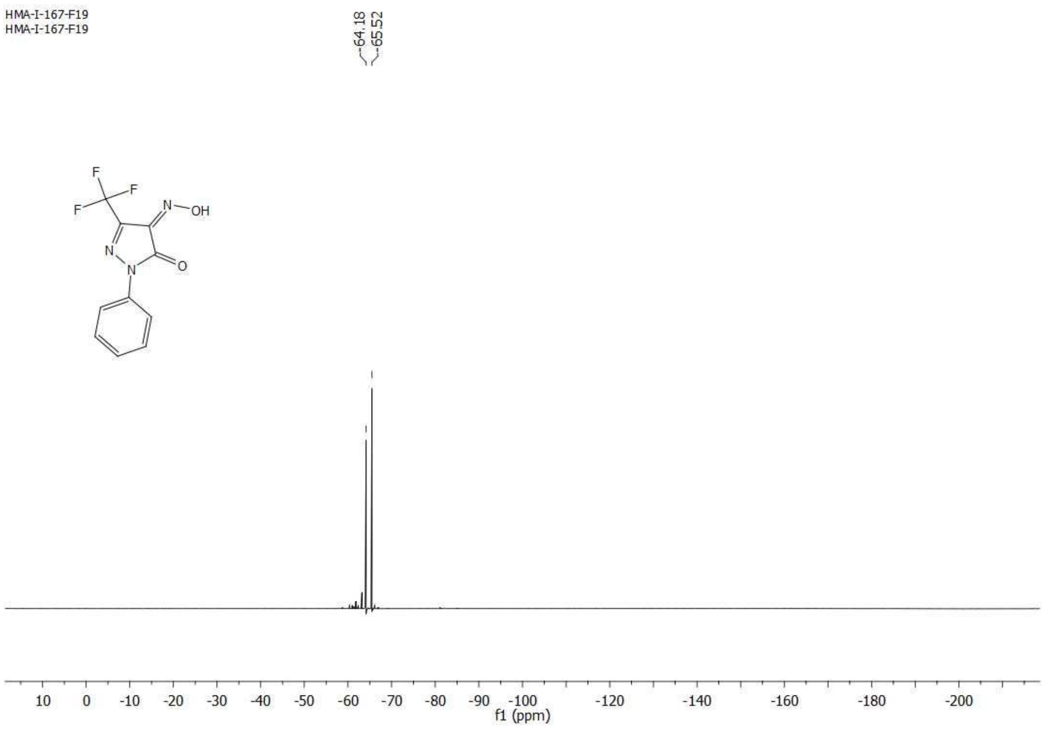

Figure S23: ^{19}F-NMR spectrum of compound **18**.

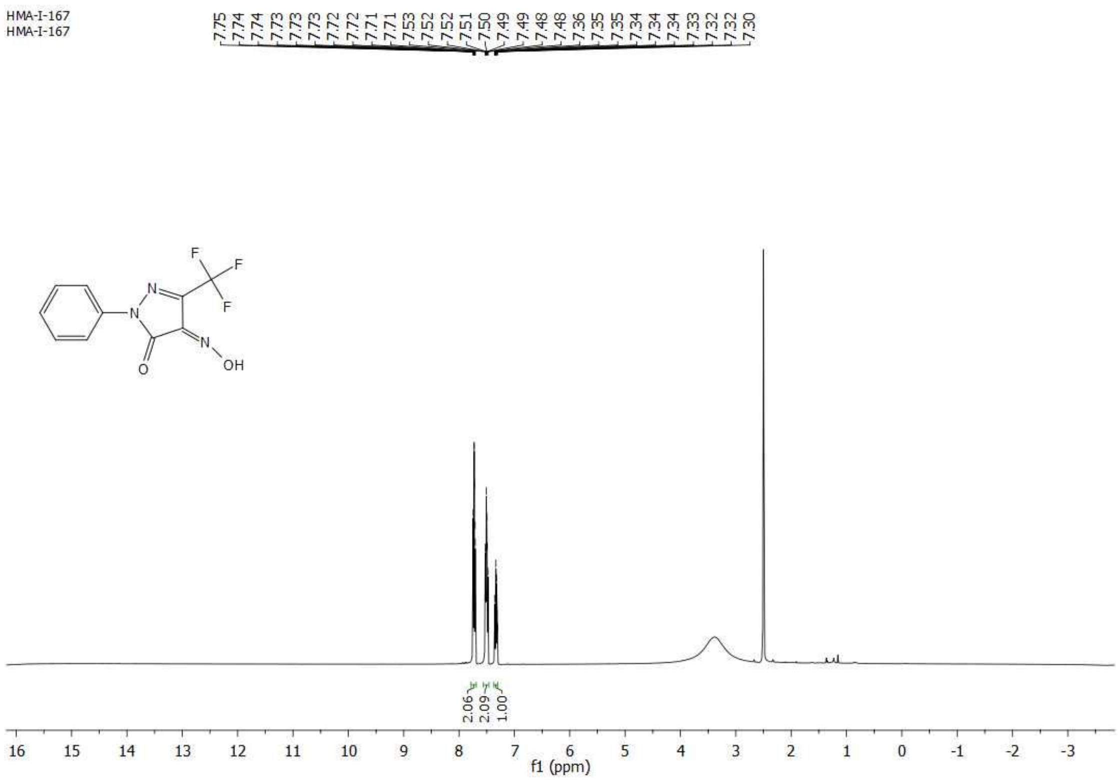

Figure S24: ^{1}H-NMR spectrum of compound **18**.

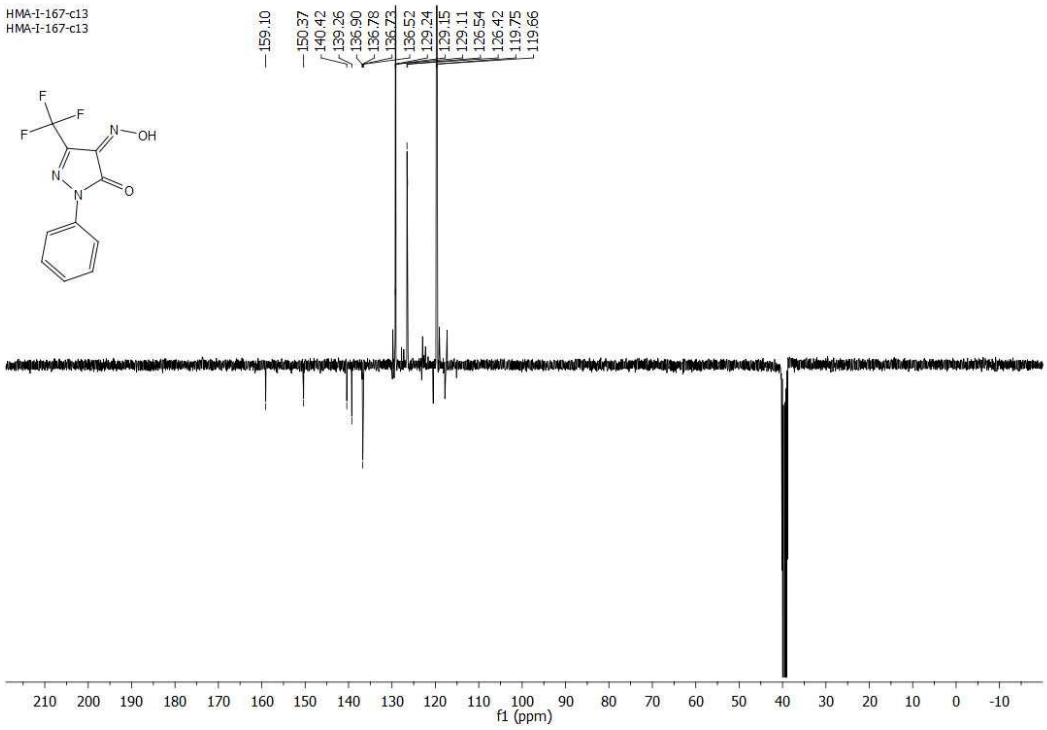

Figure S25: ^{13}C-NMR spectrum of compound **18**.

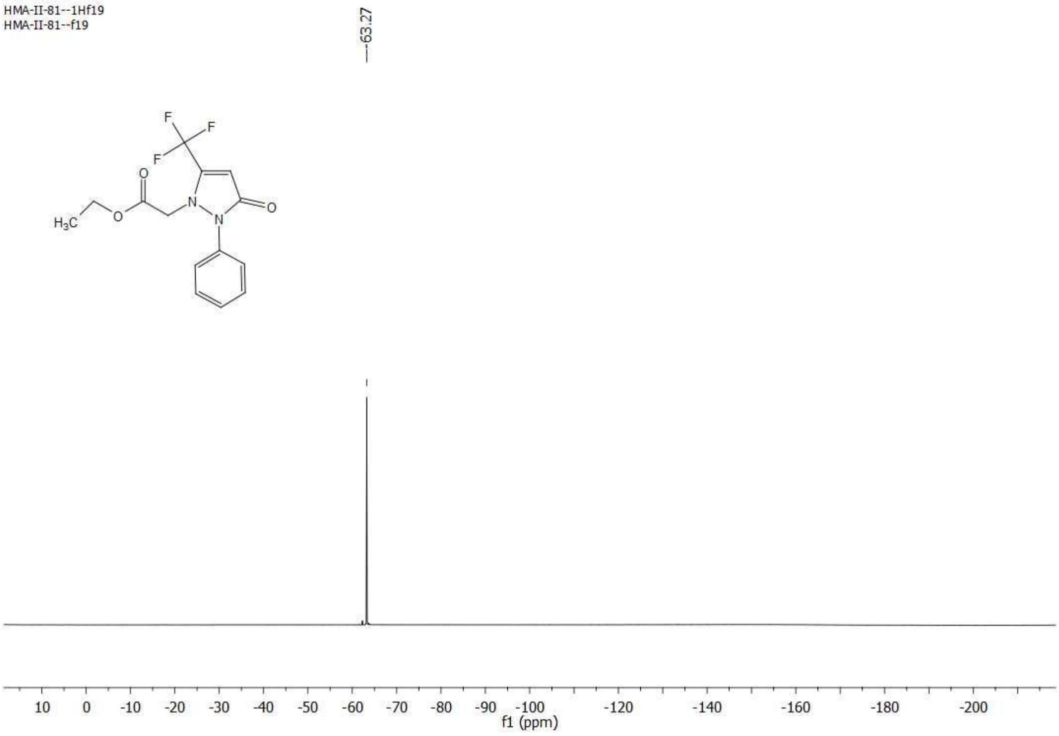

Figure S26: ^{19}F-NMR spectrum of compound **19**.

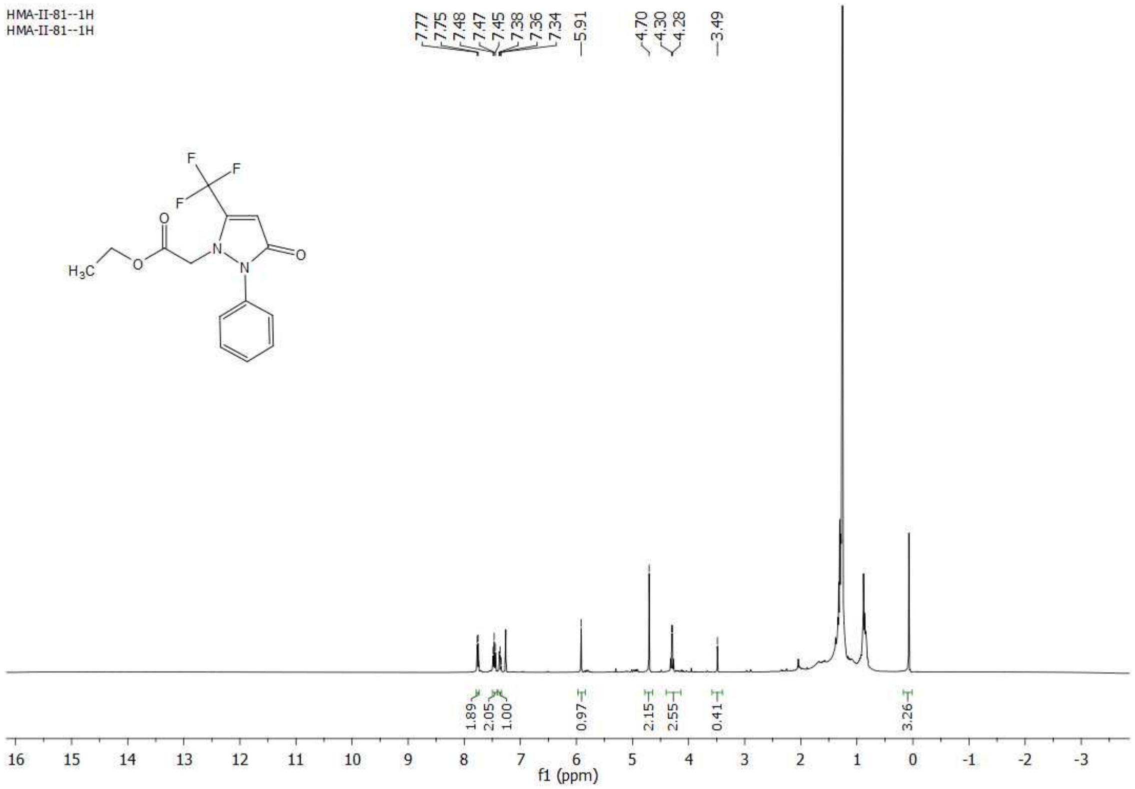

Figure S27: ^{1}H-NMR spectrum of compound **19**.

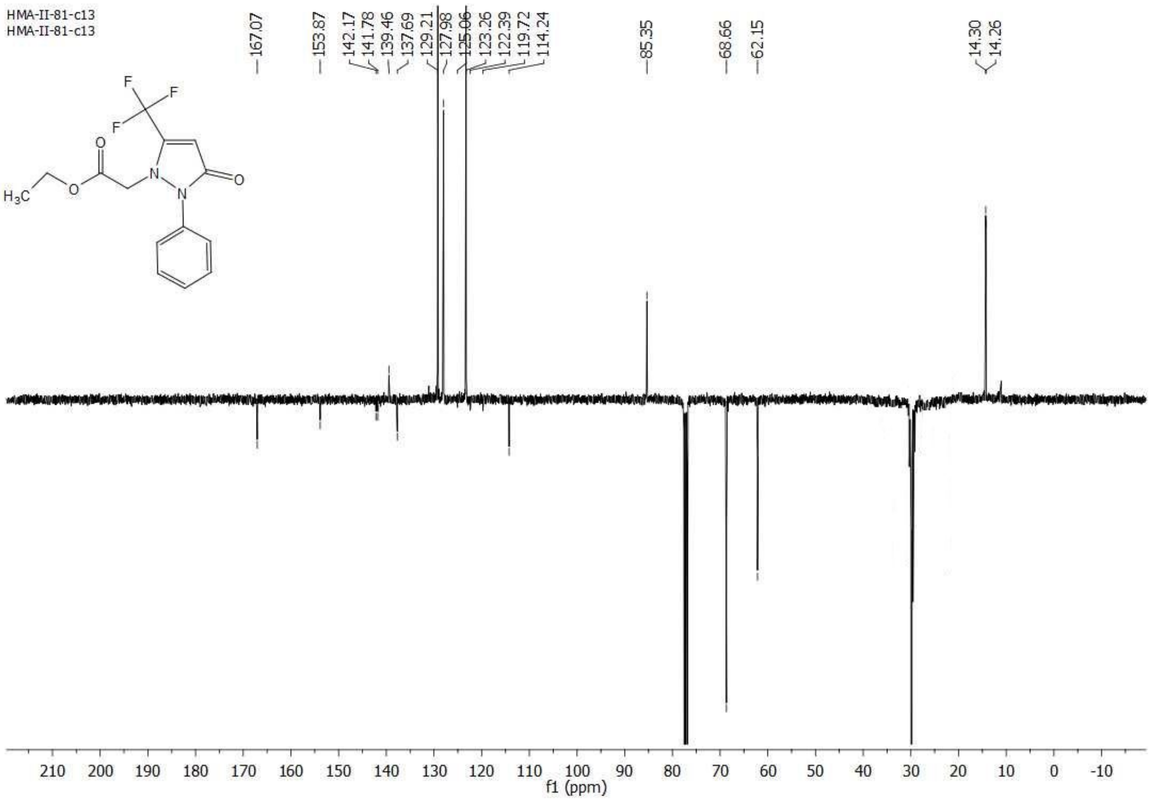

Figure S28: ^{13}C-NMR spectrum of compound **19**.

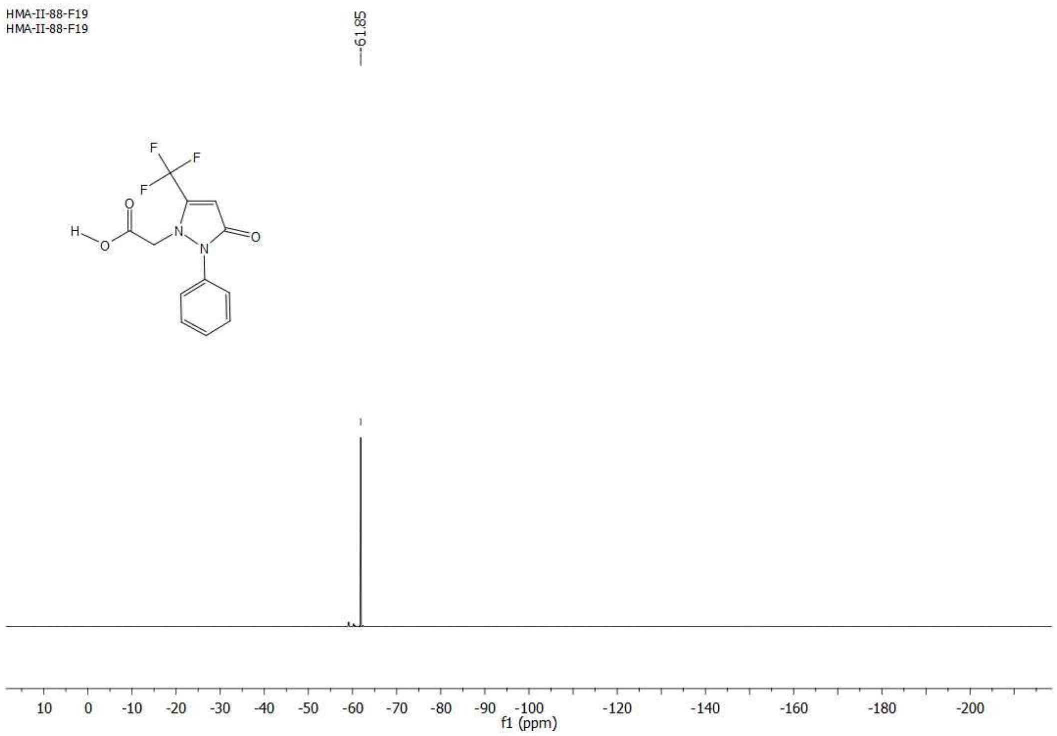

Figure S29: ^{19}F-NMR spectrum of compound **20**.

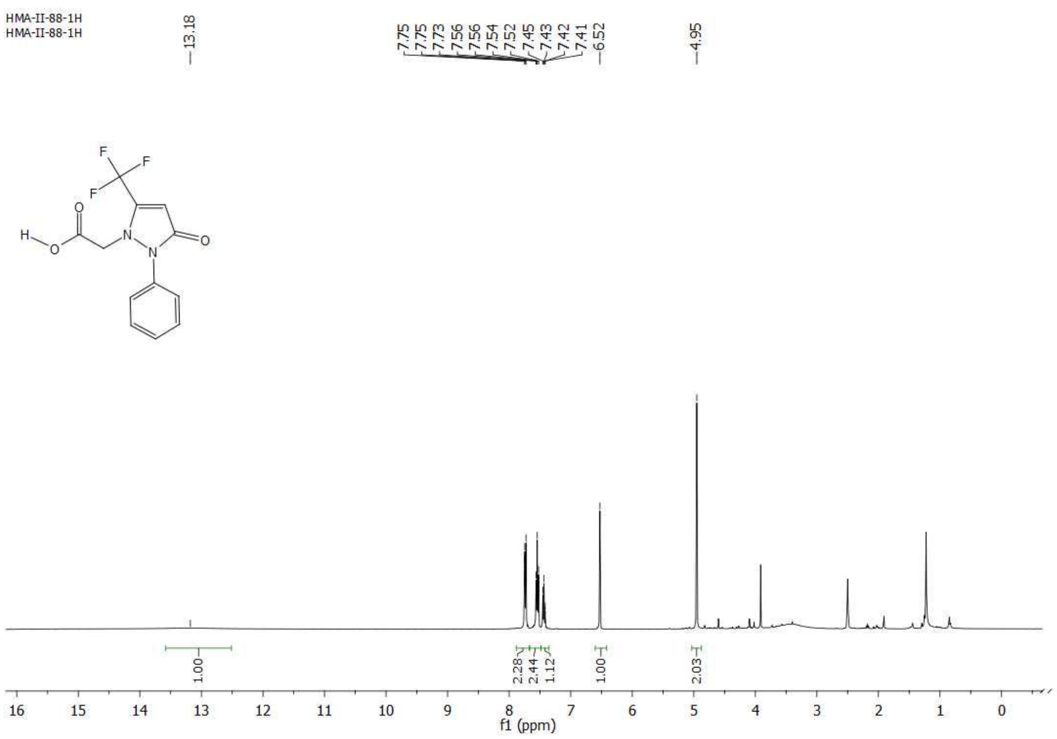

Figure S30: ^{1}H-NMR spectrum of compound **20**.

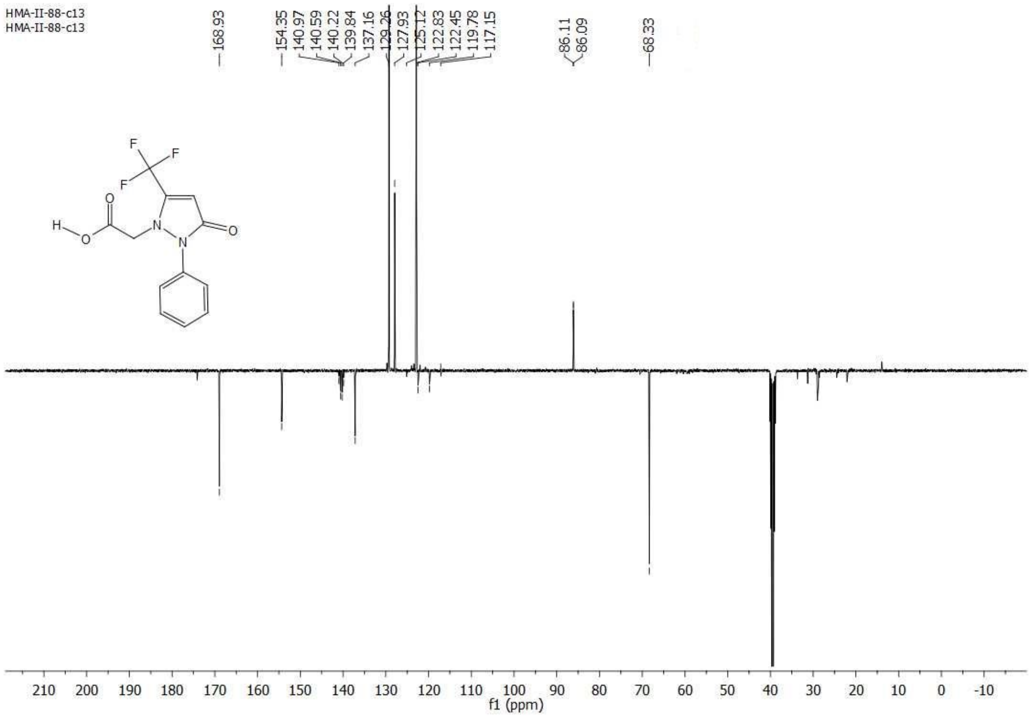

Figure S31: ^{13}C-NMR spectrum of compound **20**.

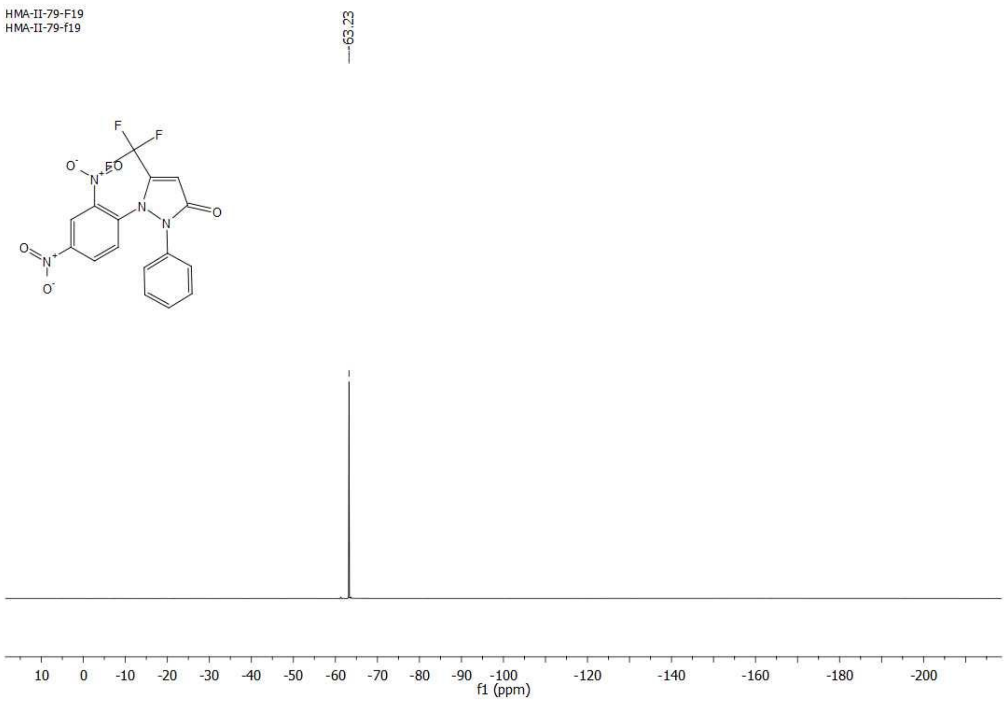

Figure S32: ^{19}F-NMR spectrum of compound 21.

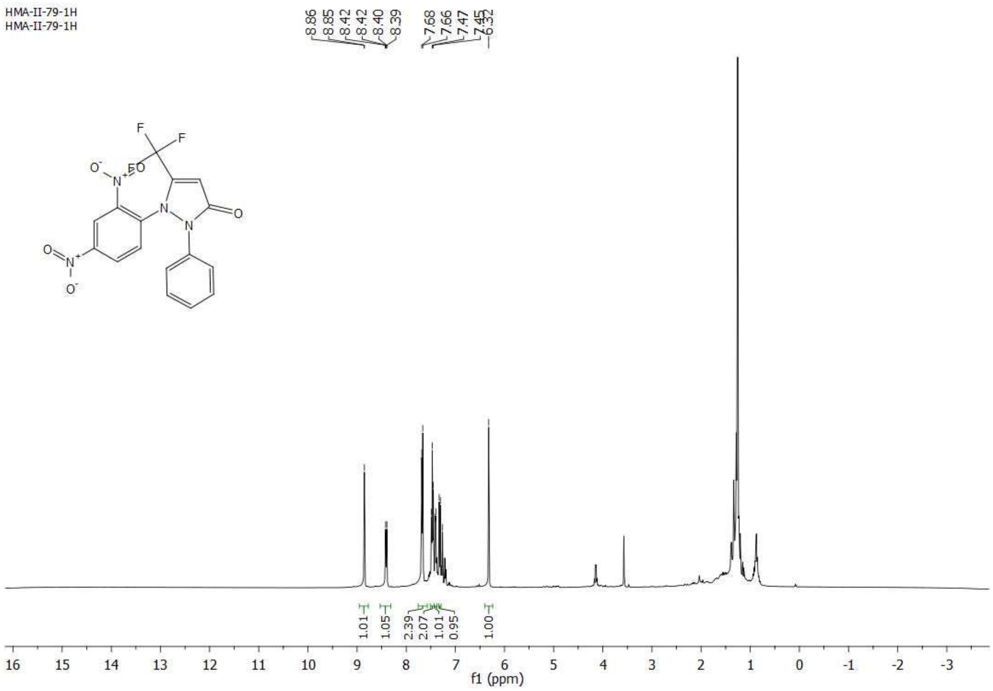

Figure S33: ^{1}H-NMR spectrum of compound **21**.

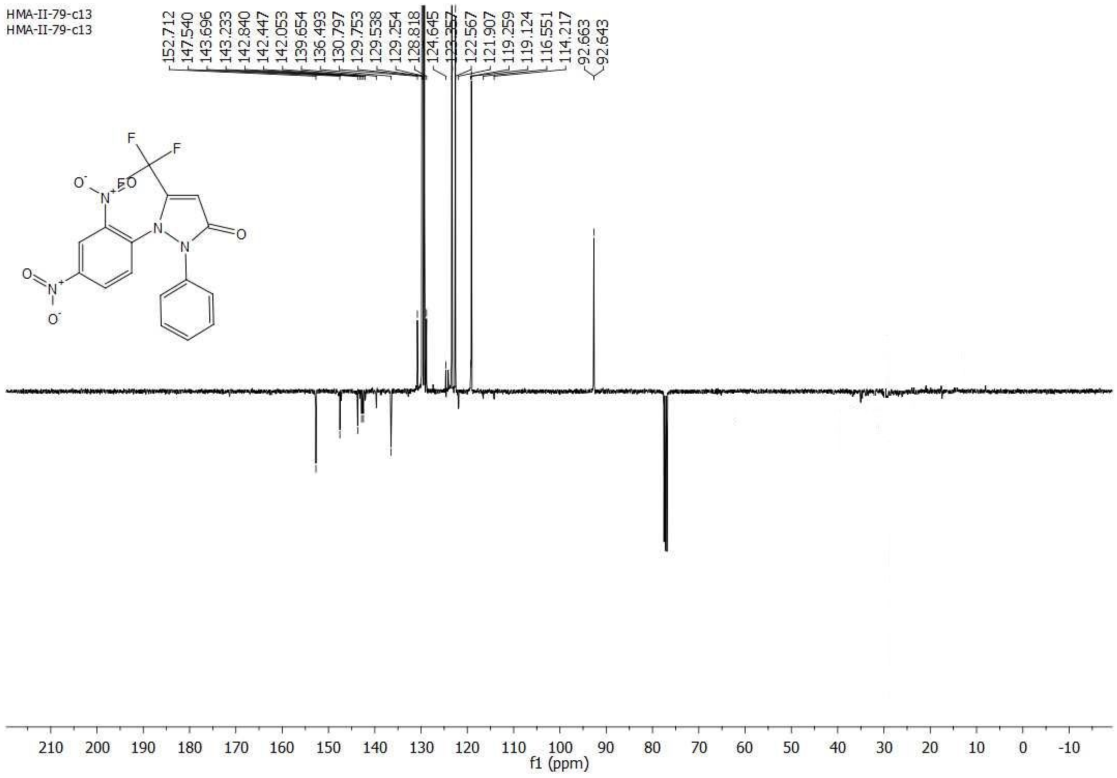

Figure S34: ^{13}C-NMR spectrum of compound **21**.

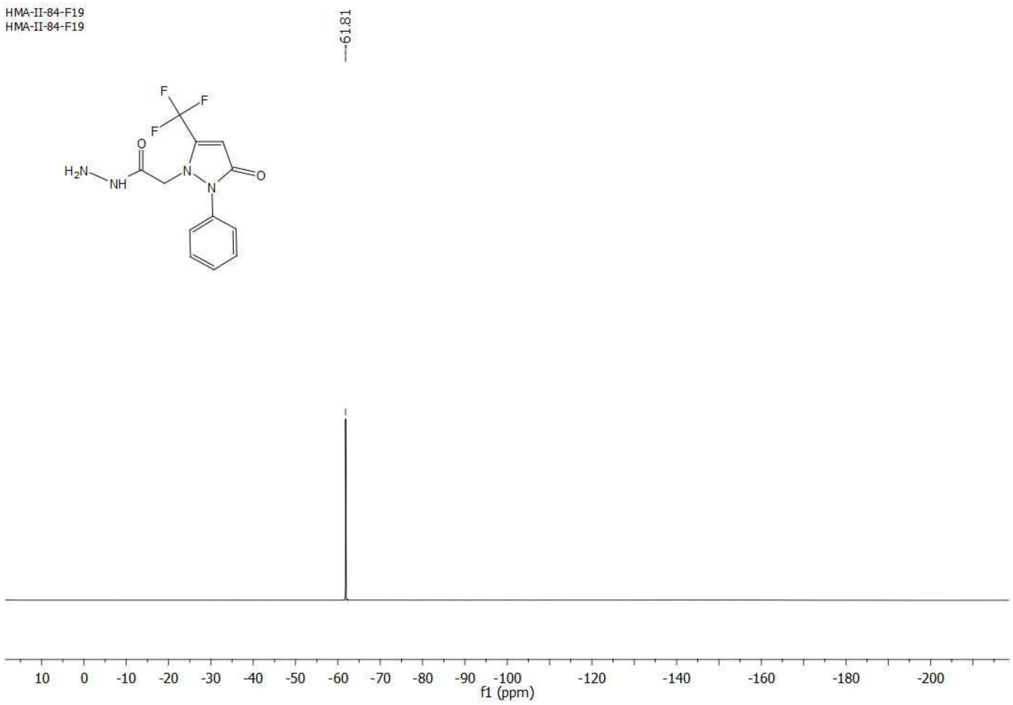

Figure S35: ^{19}F-NMR spectrum of compound **22**.

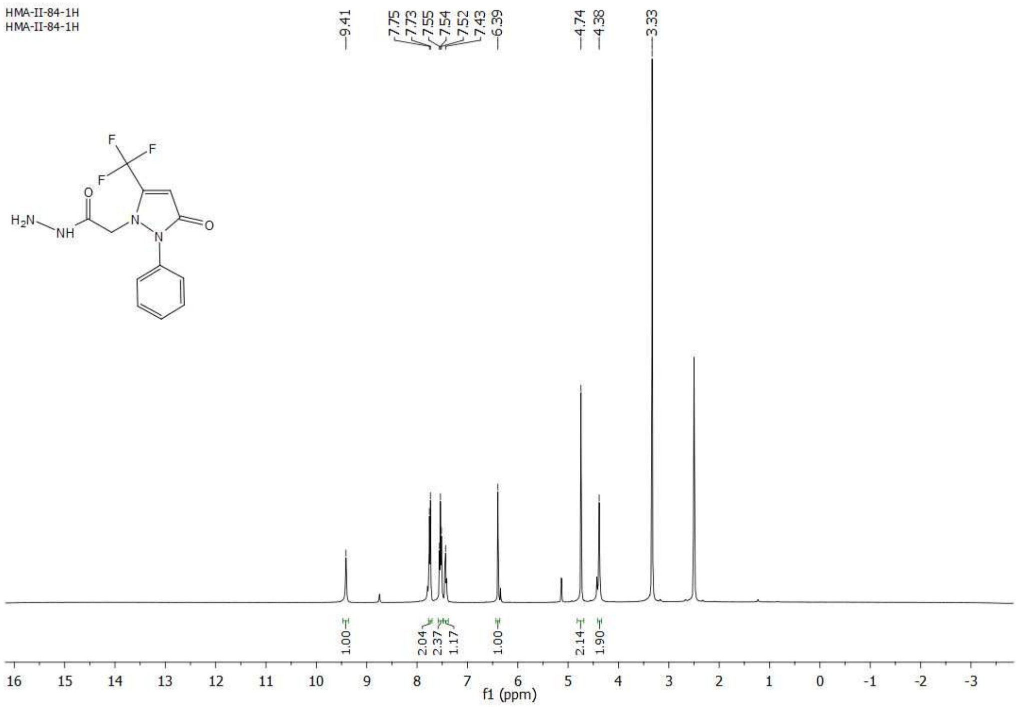

Figure S36: ^{1}H-NMR spectrum of compound **22**.

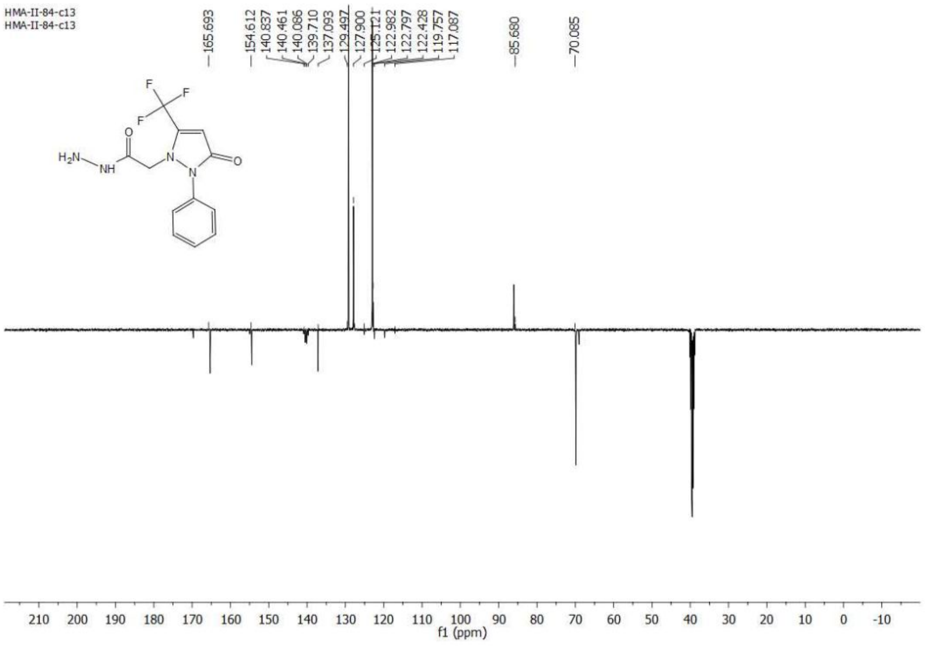

Figure S37: ^{13}C-NMR spectrum of compound **22**.

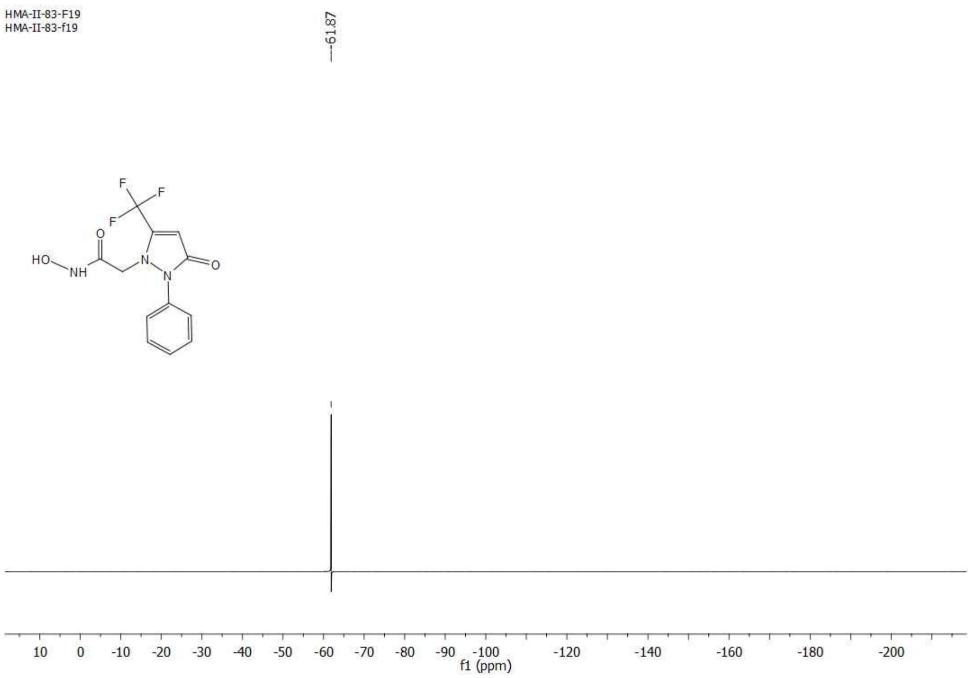

Figure S38: ^{19}F-NMR spectrum of compound **23**.

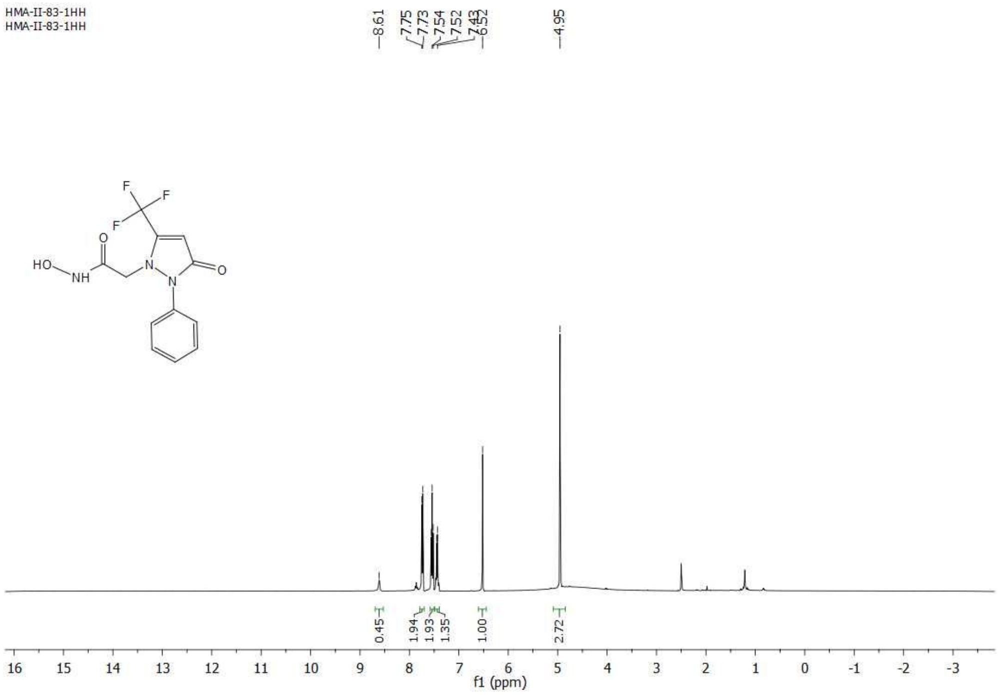

Figure S39: ^{1}H-NMR spectrum of compound **23**.

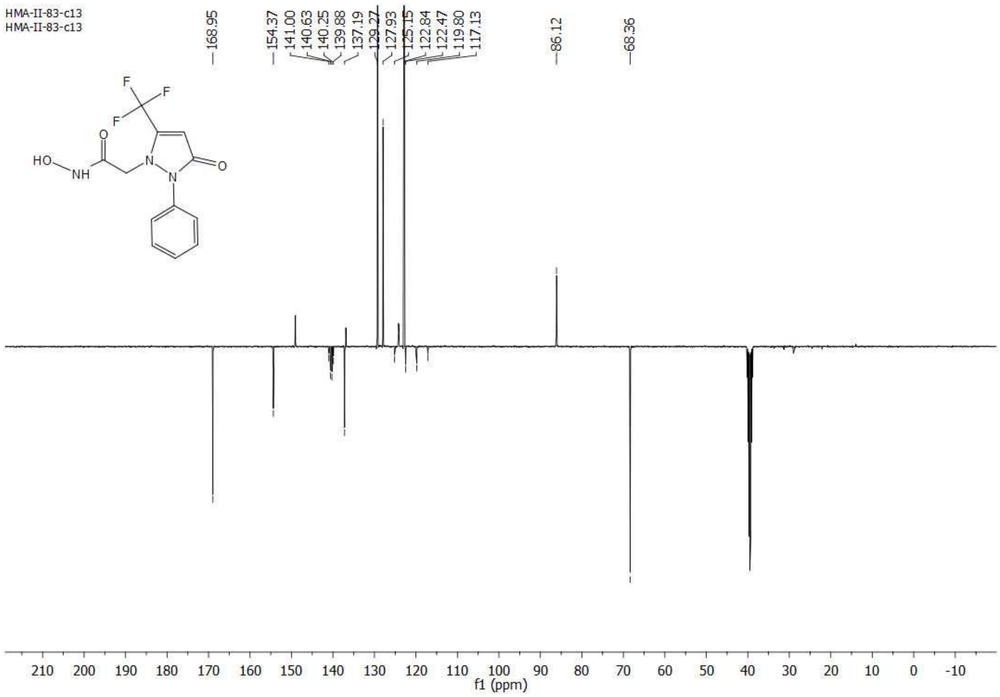

Figure S40: ^{13}C-NMR spectrum of compound **23**.

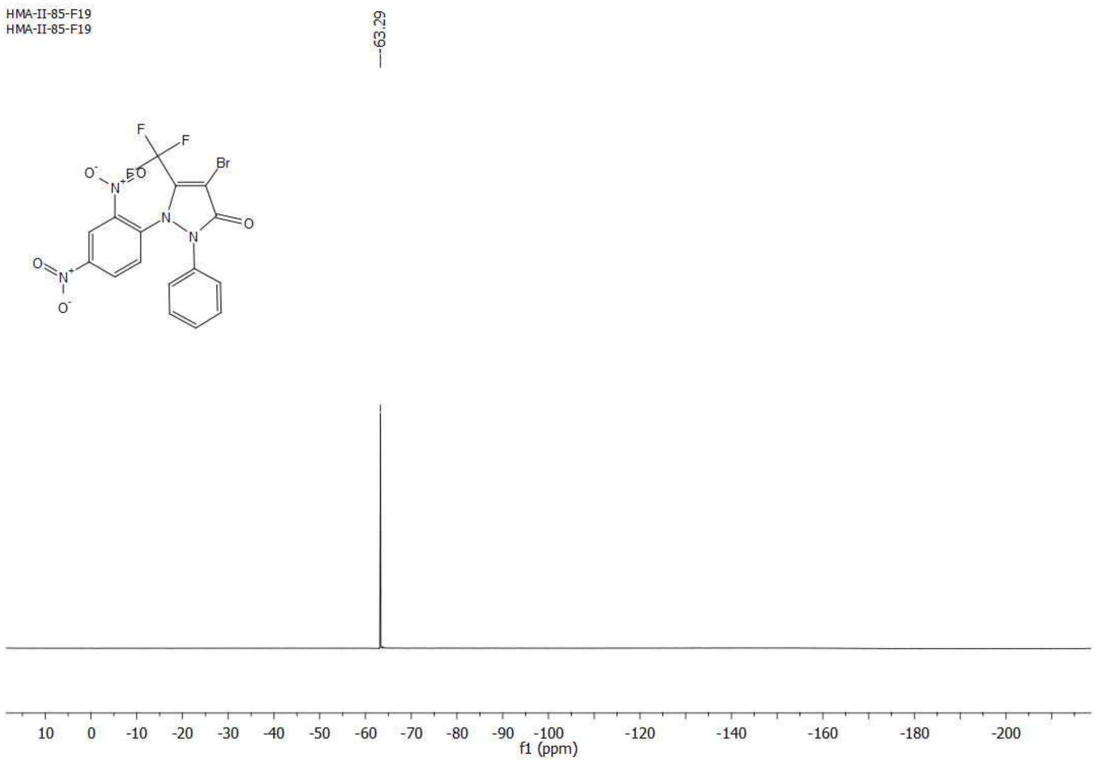

Figure S41: ^{19}F-NMR spectrum of compound **24**.

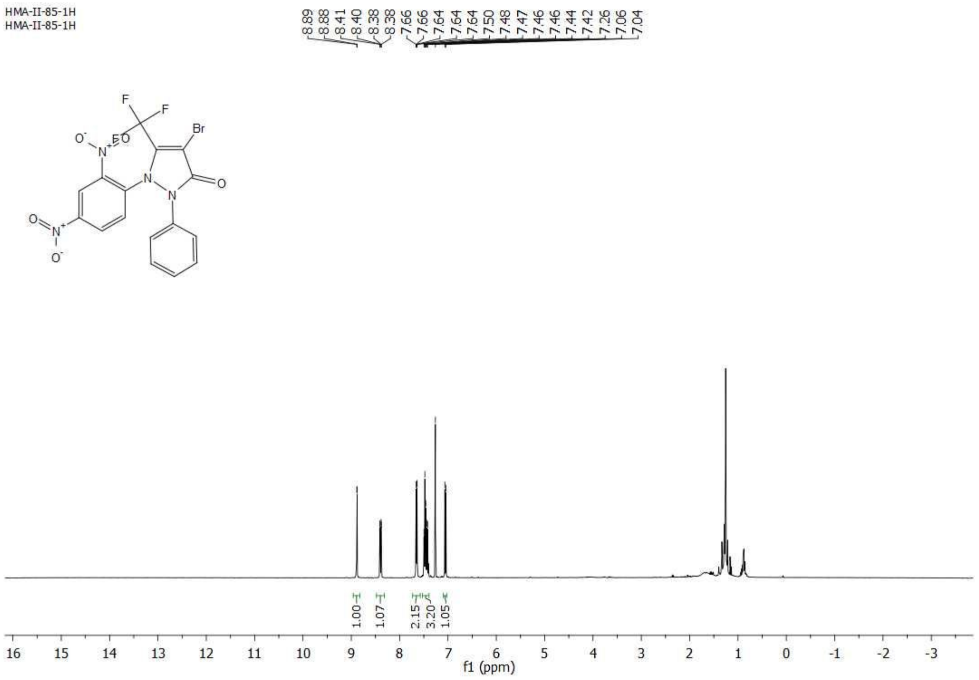

Figure S42: ^{1}H-NMR spectrum of compound **24**.

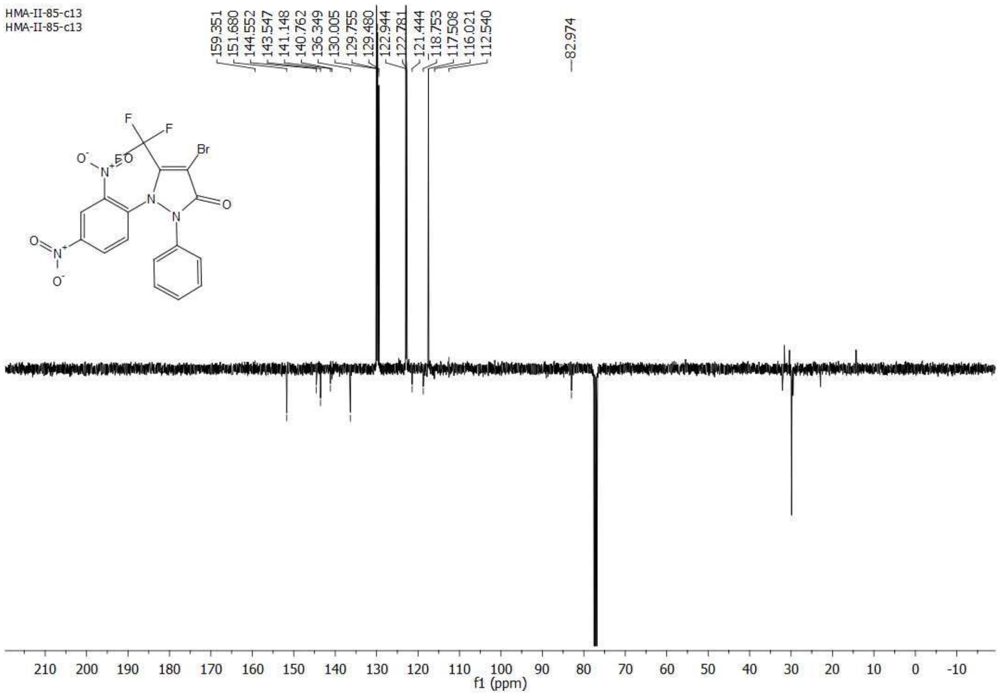

Figure S43: ^{13}C-NMR spectrum of compound **24**.

S2.1. Biological studies

Monacha cartusiana is a terrestrial habitat, breathing through a lung, with a right-coiled, smooth, thin, semitransparent, flattened, creamy or milk-white, diameter 10-12 mm, and height 6.5-7.3 mm shell. Belongs to: Order Stylommatophora (tentacles with eyes at tips), Family Hygromiidae, (hairy or smooth shells, often with an umbilicus). *Monacha cartusiana* snails were collected from the field of Mesalmia village, Hehia district, Sharkia governorate, maintained under laboratory conditions for two weeks prior to the tests, and fed daily with lettuce leaves for acclimatization. A series of concentrations, that is, four concentrations of each compound (1, 2, 3, and 4 mg/mL), were prepared by mixing an appropriate amount of each compound with 100 ml of Tween 80 and 100 ml of EtOH until the compounds became completely soluble, followed by the addition of the appropriate volume of water to provide homogeneous suspension. Before starting the experiment, the snails were starved for 48 h. The snails were irradiated with water for activation. Thirty snails for each concentration were immersed in the corresponding solution of the tested compound for 30 s three times. Every ten snails were introduced in a box supplied with a disc of lettuce. The boxes were covered with a muslin cloth and secured with a rubber band to prevent the snails' escape. Ten snails were utilized as a control by immersing them in water containing 100 ml of Tween 80 and 100 ml of EtOH.

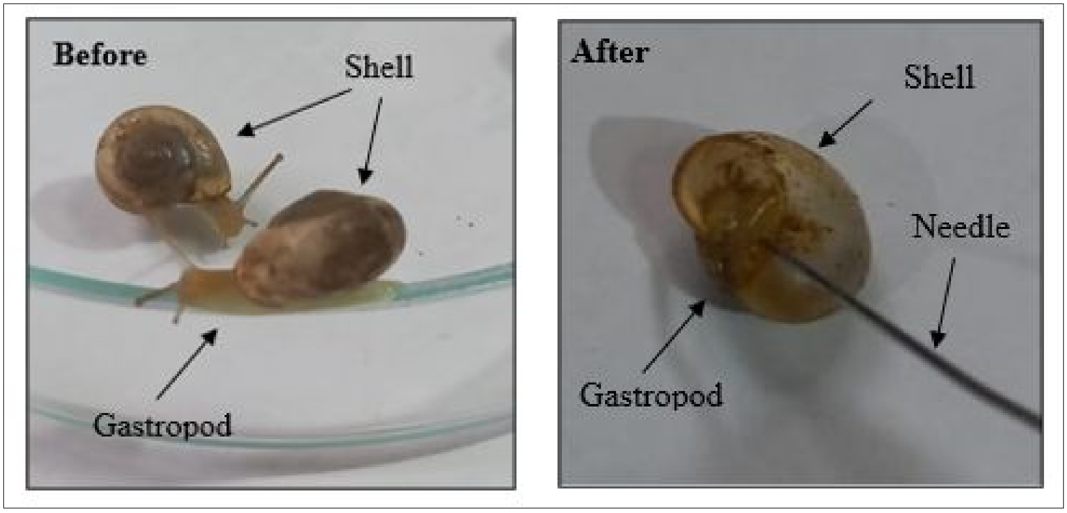

Figure S44: Representing *M. cartusiana* land snails before and after treatment.

S2.2. Biochemical assays

S2.2.1. Preparation of samples for biochemical assay

Samples used for biochemical assays were collected at 24 hrs. post treatment of LC50 for **11, 16, 17**, and **18** compounds. Untreated snails were used as control. Samples were homogenized in distilled water using a teflon homogenizer. The homogenates were centrifuged at 5000 rpm for 10 minutes at 5 ºC, the supernatants were immediately assayed to determine the level of aspartate aminotransferase (AST), alanine aminotransferase (ALT), acetylcholinesterase (AChE), total carbohydrates, and total lipids.

S2.3. Histological Study

S2.3.1 Preparation of samples for histological Study

After treating land snails with trifluoromethyl pyrazolone compounds **11, 16, 17**, and **18** with a concentration equal to the LC_{50} values, histological studies were performed. The shell of each snail was carefully broken, and the soft tissues were dissected out. The digestive gland and the intestines of the snail were placed in a petri dish containing isotonic buffer. The digestive gland was separated and fixed in 10% formalin. For histological studies, the organ was dehydrated using an ascending series of ethanol alcohol, cleared in xylene for 2 min, and then immersed in three changes: The first consisted of xylene + wax in ratio 1:1, and the second and the third are wax each for 1/2 h. Embedding in paraffin and blocking was carried out under vacuum. Serial transverse sections of 6–8 μm were mounted on clean slides without using any adhesive material. Ehleish's haematoxylin and eosin were employed for general histological studies of the digestive gland.

3. Computational Models & Docking Studies

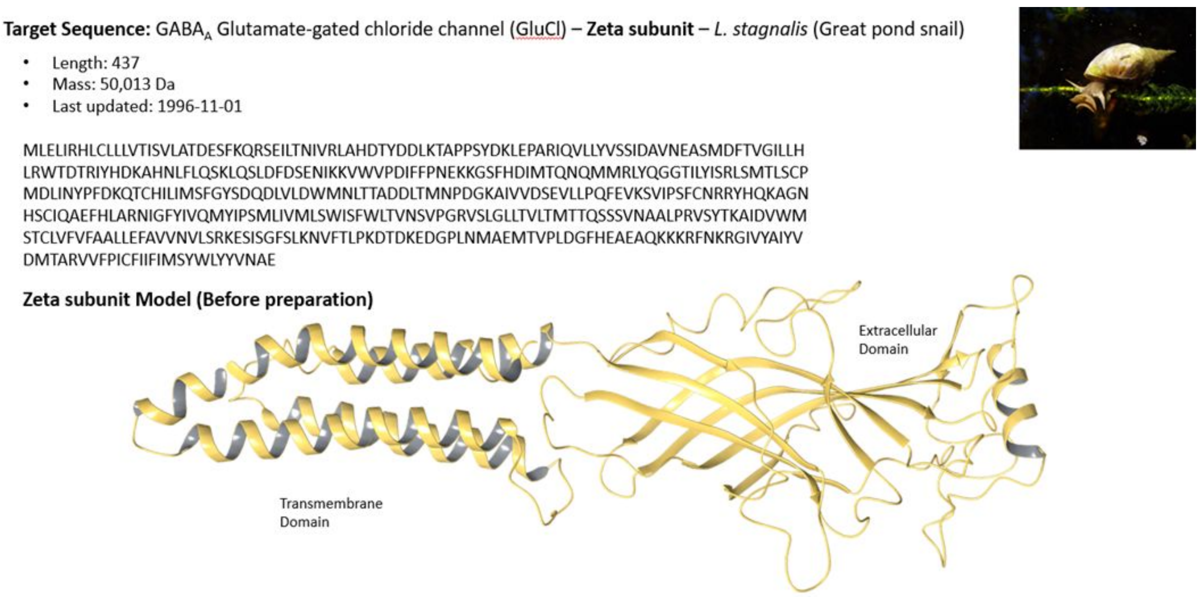

Figure S46. Target sequence of the glutamate-chloride (GluCl) zeta-subunit of the *L. stagnalis* (greant pond snail) $GABA_A$ receptor used for homology modelling. Ribbon diagram of the structure of the generated model, highlighting the transmembrane/extracellular domains.

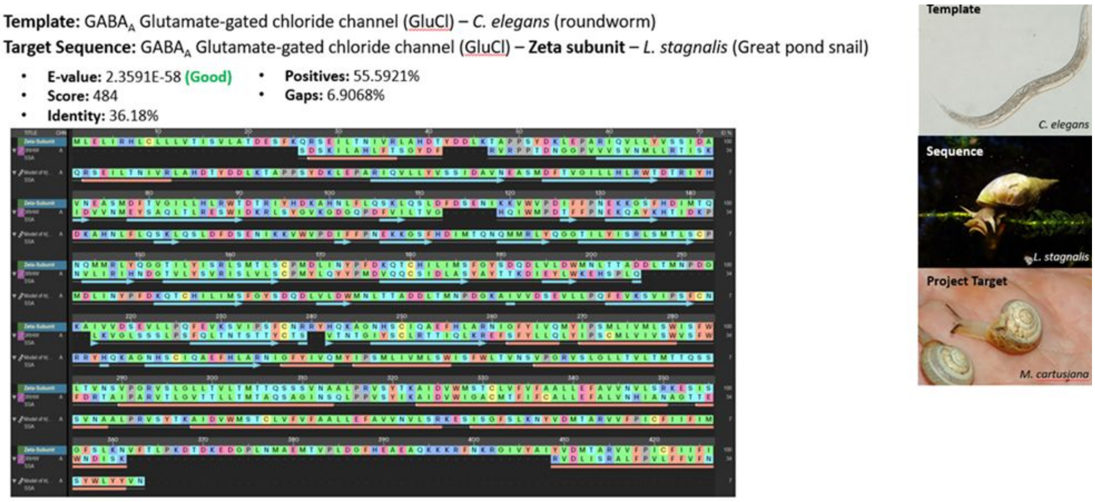

Figure S47. Homology modelling strategy showing the *C. elegans* template (GluCl $GABA_A$), and *L. stagnalis* target (zeta). Protein sequence alignment is also highlighted along with several model metrics (E-value = 2.3591 x 10^{-58}, score = 484, identity = 36.18%, positives = 55.5921%, gap = 6.9068%).

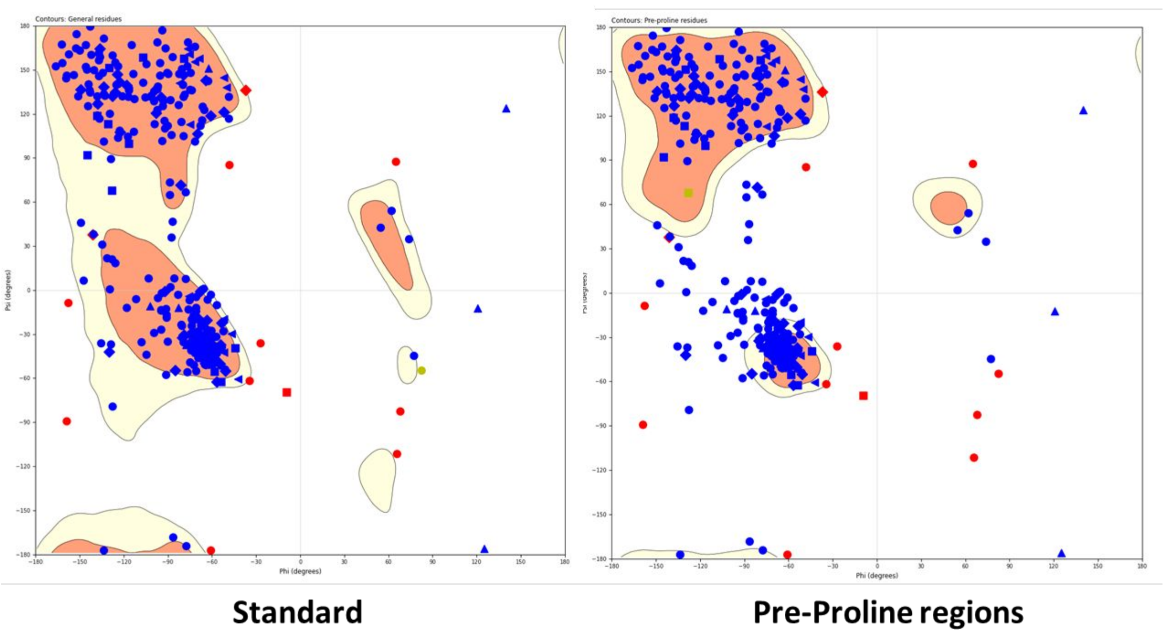

Figure S48. Ramachandran plots of the 3RHW-zeta subunit homology model using standard forbidden regions and the pre-proline residues. >95% of residues were found in the allowed regions highlighting successful model generation.
